# High-Ionic-Strength Wastewater Treatment via Catalytic Wet Oxidation over a MnCeO_*x*_ Catalyst

**DOI:** 10.1021/acscatal.2c01952

**Published:** 2022-06-13

**Authors:** Xiaoxia Ou, Helen Daly, Xiaolei Fan, Simon Beaumont, Sarayute Chansai, Arthur Garforth, Shanshan Xu, Christopher Hardacre

**Affiliations:** † Department of Chemical Engineering, School of Engineering, The University of Manchester, Oxford Road, Manchester M13 9PL, U.K.; ‡ Department of Chemistry, University of Durham, South Road, Durham DH1 3LE, U.K.

**Keywords:** high-ionic-strength wastewater treatment, phenol, catalytic wet oxidation (CWO), MnCeO_*x*_, in situ ATR spectroscopy

## Abstract

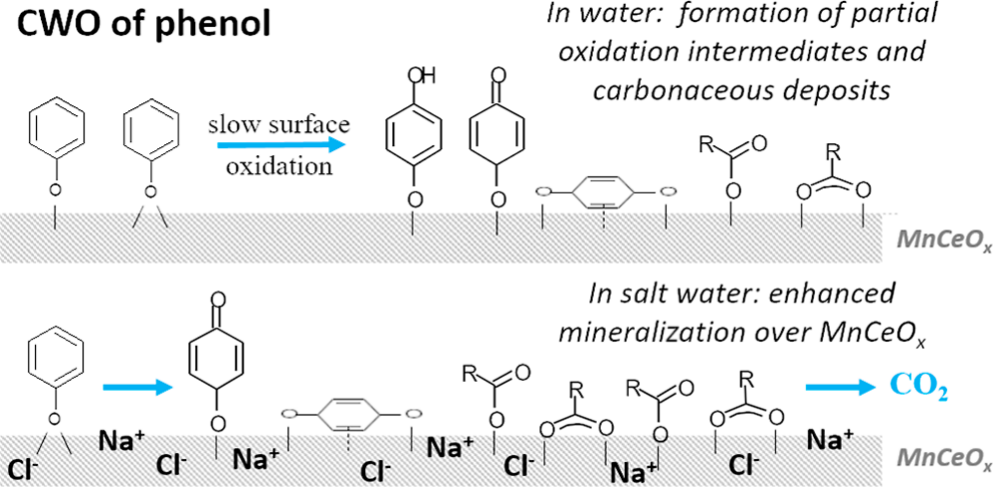

Catalytic wastewater treatment has rarely been applied to treat high-ionic-strength wastewater (HISWW) as it contains large amounts of catalyst poisons (e.g., Cl^–^). This work investigates the catalytic wet oxidation (CWO) of phenol over a MnCeO_*x*_ catalyst in the presence of high NaCl concentrations where the combination of MnCeO_*x*_ and NaCl promoted the CWO of phenol. Specifically, in the presence of NaCl at a concentration of 200 g L^–1^ and MnCeO_*x*_ at a concentration of 1.0 g L^–1^, phenol (initially 1.0 g L^–1^) and total organic carbon (TOC) conversions were ∼98 and 85%, respectively, after a 24 h reaction. Conversely, under the same reaction conditions without NaCl, the catalytic system only achieved phenol and TOC conversions of ∼41 and 27%, respectively. In situ Attenuated Total Reflection infrared spectroscopy identified the nature of the strongly adsorbed carbon deposits with quinone/acid species found on Ce sites and phenolate species on Mn sites in the single oxides and on MnCeO_*x*_. The presence of high concentrations of NaCl reduced the carbon deposition over the catalyst, promoting surface oxidation of the hydrocarbon and reoxidation of the catalyst, resulting in enhanced mineralization. Moreover, the used MnCeO_*x*_ catalyst in the salt water system was efficiently regenerated via a salt water wash under the reaction conditions, showing the great potential of MnCeO_*x*_ in practical HISWW treatment.

## 1 Introduction

Shale gas extraction is one route to meeting the energy demands of modern society. However, although it has enhanced the global gas production, bridging the current gap between nonrenewable (81.6%) and renewable (13.4%) energy sources,^1^ it comes with significant environmental impact. One major impact is the large amounts of fresh water required for shale gas extraction, which leads to the discharge of high-ionic-strength wastewater (HISWW) in the form of produced waters, for example, hydraulic fracturing demands of as much as 34,900 m^3^ of water per horizontal well.^2^ In addition to the large quantity, HISWW is also highly contaminated, making it difficult to treat.

Produced water contains various inorganic and organic contaminants, such as oils, organic acids, and phenolics, and has high salinity and hardness.^3^ Biological, physical, and chemical treatments have been applied to treat produced water, including activated sludge, adsorption, distillation, filtration, precipitation, and oxidation technologies.^4^ Among these technologies, special emphasis has been placed on using advanced oxidation processes (AOPs), which have the ability to mineralize refractory compounds in a relatively short treatment time.^3^

Conventional heterogeneous catalysts used for AOPs are typically composed of metals supported on oxides, for example, Pt/TiO_2_.^5^ However, it is well-understood that halide species present in wastewaters can strongly poison the metal sites in oxidation catalysts, resulting in rapid catalyst deactivation,^6^ and relevant research using such supported metal catalysts for HISWW treatment is rare. For example, Pt-based membrane reactors have been demonstrated to be effective for the wet air oxidation of organics (e.g., formic acid and 2,4-dimethyl phenol) in water and sea water; however, the catalytic system exhibited low reaction rates in the presence of halide compounds due to halide poisoning of the Pt sites.^7^ Although the spent catalyst could be regenerated via a fresh water wash, the levels of halide tested by relevant studies, to date, are relatively low (0.15 g L^–1^) compared with the concentrations that are found in HISWW. For example, the Cl^–^ concentration in produced water can commonly be up to 200 g L^–1^.^8,9^

Solid oxides can be employed as halide-resistant catalysts, such as MnCeO_*x*_,^10^ OMS-2,^11^ and CeO_2_–TiO_2_.^12^ MnCeO_*x*_ has been used in the oxidation of chlorinated organics (e.g., chlorobenzene and dichlorophenol), showing resistance to Cl^–^,^10^ and no chlorinated-species-promoted leaching was observed.^13^ This catalyst has also been used for wet air oxidation of aniline, where addition of HCl reduced the metal leaching of the catalyst, giving rise to a stable performance for over 200 h.^14^ Catalytic wet oxidation (CWO) is an efficient AOP for the treatment of wastewater with medium/high loads of refractory organic pollutants, with a number of industrial CWO plants in operation.^15^ Industrial CWO processes treating wastewater typically operate at temperatures above 160 °C, often utilizing noble metal-based catalysts that can be poisoned by Cl^–^, hence limiting their application in the treatment of high-salinity wastewater.^16^

In this study, CWO using MnCeO_*x*_ catalysts for the treatment of phenol in water with high NaCl concentrations of up to 200 g L^–1^ has been investigated under mild reaction conditions. Phenol was chosen as the model organic pollutant due to its significant contribution to the toxicity of HISWW,^17^ as well as its common presence (0.009–23 mg L^–1^).^9,18^ MnCeO_*x*_ was employed as the catalyst due to its high oxidation efficiency and good resistance to leaching and halide poisoning.^19−21^ In this system, the CWO activity has been attributed to the synergistic effect between manganese and cerium oxides, which improves the oxygen storage capacity and oxygen mobility on the catalyst surface, leading to higher catalytic activity than that of the single oxides.^19,22^

The wet oxidation of phenol over MnCeO_*x*_ catalysts has been reported to occur via a reactive adsorption mechanism^20^/dual-site Langmuir–Hinshelwood reaction path.^21^ The catalytic pathway involves rapid initial adsorption of phenol on the catalyst, followed by oxidation to intermediates or mineralization to CO_2_ and replenishment of oxygen to the catalyst with undesired side reactions including the formation of byproducts and carbon deposition proceeding in parallel.^21^ However, mineralization is the rate-limiting step of CWO of phenol on MnCeO_*x*_ catalysts as surface oxidation is much slower than adsorption. The adsorption of phenol and the intermediates in the phenol oxidation pathway on the surface of the catalyst, namely, the coverage and nature of the deposit, can determine the extent of its subsequent oxidation. Delgado et al.^23^ reported that phenol is fully converted into carbonaceous deposits and the oxidation of the carbonaceous deposits occurred as the initial step for the total mineralization of phenol at moderate temperatures (130–160 °C) and oxygen partial pressures (1–2 MPa), while being the origin of deactivation for phenol removal at milder conditions (temperatures below 110 °C), at which complete deactivation was eventually obtained. The adsorbed carbon species have been proposed to be hydrocarbon-like in nature (i.e., oxidative polymerization of phenol) and identified as aromatic and carboxylic acid functionalities using X-ray photoelectron spectroscopy (XPS) and ^13^C NMR.^24^ Despite the carbon deposited on the surface of MnCeO_*x*_ playing an important role in phenol oxidation,^25^ to date, much of the literature on CWO of phenol lacks clarity regarding the roles of Mn and Ce sites in the reactive adsorption/oxidation of the adsorbed carbon species. The presence of Mn has been reported to favor the formation of the carbonaceous deposit, with Ce proposed as being more active in the oxidation to CO_2_ and H_2_O,^23^ while Chen et al.^19^ suggested that the carbonaceous deposition mainly occurred on the cerium-related sites. With the adsorption of phenol on the catalyst and the nature of the carbon deposits formed identified to be key in the mineralization ability of the catalysts, in situ ATR spectroscopy was utilized in this study to probe the adsorption/oxidation of phenol over MnCeO_*x*_ in the presence and absence of a high concentration of NaCl.

## 2 Experimental Section

### 2.1 Chemicals and Catalyst Preparation

Manganese(II) nitrate tetrahydrate [Mn(NO_3_)_2_·4H_2_O] was obtained from Alfa Aesar. Cerium(III) nitrate hexahydrate [Ce(NO_3_)_3_·6H_2_O] was obtained from Aldrich. Sodium hydroxide (NaOH) was obtained from Honeywell. Phenol (C_6_H_5_OH) was obtained from Sigma-Aldrich. All the chemicals were used as received.

MnCeO_*x*_ catalysts were prepared using the coprecipitation method, and excess NaOH was added in aqueous solutions of Mn(NO_3_)_2_ and Ce(NO_3_)_3_ (theoretical Mn/Ce atomic ratio = 1.5). The precipitate was centrifuged to remove excess liquid and washed with water till the pH of water after wash was no longer changed. The product was calcined in a muffle furnace at 400 °C (ramp at 5 °C min^–1^) for 6 h.

### 2.2 Catalyst Characterization

Fresh and used MnCeO_*x*_ catalysts were characterized using a number of techniques. Catalyst powders were deposited onto a clean silicon wafer for X-ray diffraction (XRD) tests, and their patterns were obtained using a Philips X’Pert X-ray diffractometer (Cu Kα_1_ radiation, λ = 1.5406 Å, 40 kV, 40 mA, 5° < 2θ < 90°, 0.0167° step size). Fresh MnCeO_*x*_ powders were sonicated in ethanol and placed onto a stub using double-sided carbon tape for scanning electron microscopy (SEM) with energy-dispersive X-ray spectroscopy (EDS) analysis. The morphology of the catalysts was acquired using a Quanta 250 (beam acceleration voltage = 20 kV), and quantitative element analysis was carried out using point scan and mapping (manufacturer inbuilt calibration was applied). Mn and Ce contents of the samples were also determined using a PANalytical MiniPal 4 (PANalytical EDXRF) spectrometer. N_2_ physisorption analysis at −196.15 °C was performed using a Micromeritics ASAP 2020 analyzer. Before N_2_ sorption analysis, the samples were degassed at 300 °C for 4 h. The specific surface area of the materials was determined using the Brunauer–Emmett–Teller (BET) method. XPS analysis was conducted on a Kratos AXIS Ultra DLD apparatus equipped with a monochromated Al Kα X-ray source, a charge neutralizer, and a hemispherical electron energy analyzer, and catalyst powders were deposited on a carbon-coated specimen holder before measurements. During data acquisition, the chamber pressure was kept below 10^–9^ mbar, and a pass energy of 40 eV was used. The spectra were analyzed using CasaXPS software, where fitting of the O 1s spectra was conducted with a fixed full width at half maximum value and four components using a Shirley background and correction for charging was performed using the C 1s binding energy as the reference at 284.8 eV.^26^ Temperature-programed oxidation mass spectroscopy (TPO-MS) was carried out to investigate the desorption properties of adsorbed carbon on the used catalysts. Approximately 75 mg of the used catalyst was exposed to 10% O_2_/Ar with a total flow rate of 100 cm^3^ min^–1^ for 10 min at 100 °C before the temperature was ramped up to 600 °C at a rate of 10 °C min^–1^. Desorbed CO_2_, H_2_O, and O_2_ species were monitored using a Hiden Analytical HPR20 quadrupole mass spectrometer.

### 2.3 CWO of Phenol

Synthetic HISWW samples were formulated with 1.0 g L^–1^ phenol. Different amounts of NaCl were added into the synthetic HISWW samples to mimic the high ionic strength (NaCl concentrations of 100 and 200 g L^–1^). CWO of phenol was performed in a polytetrafluoroethylene-lined Parr reactor operated in the batch mode at 110 °C and a O_2_ partial pressure of 0.5 MPa in 50 mL of water or HISWW. The MnCeO_*x*_ catalyst (different loadings in the range of 1–5 g L^–1^) was added, and the reactor was sealed and purged with N_2_ before being heated to 110 °C. At 110 °C, the reactor was pressurized with O_2_ to 0.5 MPa and agitated at 1800 rpm to initiate the reaction. This point was taken as time zero for the reaction. In addition, control experiments to probe the intrinsic oxidation ability of MnCeO_*x*_ were also performed, that is, reactions under N_2_ in water and HISWW with and without the presence of MnCeO_*x*_, as presented in Figure S1.

In all reactions, the MnCeO_*x*_ catalyst was sieved to a particle size below 95 μm, and the calculated Weisz–Prater value was 0.1, indicating that no diffusion limitations were present under these reaction conditions. In the experiment, 0.5 MPa O_2_ was used, corresponding to an O_2_/phenol stoichiometric ratio of ∼7, which was theoretically sufficient to enable the full conversion of phenol in the aqueous phase; specifically, full conversion of the phenol in the system requires 0.25 MPa O_2_. 1–2 mL water samples were collected at regular intervals over the course of the reaction. These were filtered using a syringe filter to remove the catalyst before their analysis using high-performance liquid chromatography (HPLC), total organic carbon (TOC) analysis, and inductively coupled plasma optical emission spectrometry (ICP-OES).

An HPLC system (Agilent 1220 Infinity LC system with a diode array detector emitting in the UV range with a Syncronis C18 column) was used to analyze the concentration of phenol. The HPLC conditions were as follows: UV wavelength = 270–320 nm, oven temperature = 60 °C, sample injection volume = 5 μL, and flow rate = 0.6 mL min^–1^. Mobile phases were water–acetic acid (solvent A, 99.9:0.1, v/v) and methanol–acetic acid (solvent B, 99.9:0.1, v/v). The gradient elution program was as follows: 5–95% solvent B (0–19.5 min), 95% solvent B (19.5–21 min), and 5% solvent B (21–22.5 min). Small organic acids were analyzed using an Agilent 1260 Infinity HPLC system (with Aminex HPX-87H column and UV and RI DAD detectors). The HPLC conditions were as follows: UV wavelength = 210 nm, oven temperature = 50 °C, sample injection volume = 20 μL, and flow rate = 0.6 mL min^–1^. The mobile phase was 0.005 M H_2_SO_4_. TOC analysis of the effluent was performed on a TOC analyzer (TOC-VCSH, Shimadzu). Metal leaching from the catalyst during reactions was measured using ICP-OES (PlasmaQuant PQ 9000 Elite, Analytic Jena), and the calibration curves are present in Figure S2. pH values of the water samples after reactions were analyzed using a pH meter (HI 2550, Hanna Instruments).

The used catalysts were employed in the reaction system under the same conditions for a second reaction. The initial phenol concentration in the second reaction was 1.0 g L^–1^ after the addition of more phenol into the reaction solution. Phenol conversion of the second reaction was measured using HPLC. In order to regenerate the catalyst, the following two methods were used:

(i) the catalyst was recovered from the reaction solution via centrifugation before calcination at 300 °C for 6 h or
(ii) the catalyst was recovered via centrifugation before washing with pure water (0 g L^–1^ NaCl) or salt water (200 g L^–1^ NaCl) in the Parr reactor under reaction conditions (i.e., at 110 °C and 0.5 MPa O_2_ for 2 h).

The regenerated MnCeO_*x*_ catalysts were tested under the same reaction conditions as those described above to test their catalytic activity in CWO and assess the viability of the different regeneration procedures. Small mechanical losses of the catalyst as a result of the recovery or liquid removal from the reactor in the washing procedures could occur, and therefore, complete recovery of activity in the recycle reactions would not be expected.

In this study, the change in phenol concentration in the reaction is due to the reactive adsorption on the surface of the catalyst, oxidation to intermediates in the liquid phase, and mineralization to CO_2_. The mass balance was calculated based on carbon using the unreacted phenol and converted organic carbon consisting of intermediate products in the liquid phase and the carbon adsorbed on the catalyst surface. The converted organic carbon and the organic carbon in the liquid phase were assessed through the TOC concentrations using a TOC analyzer, and the adsorbed carbon concentration was assessed using TPO-MS.

### 2.4 In Situ Attenuated Total Reflection Infrared Spectroscopy

The catalyst layers were prepared by depositing a slurry of MnCeO_*x*_ or single oxides CeO_2_ and MnO_*x*_ in water onto a ZnSe crystal and evaporating to dryness at room temperature (RT) overnight. In situ attenuated total reflection infrared (ATR-IR) spectra were recorded using a PIKE ATRMaxII accessory with an in-house modified ATR flow cell housed in a Bruker Tensor II spectrometer. The in situ ATR experiments were performed at 95 °C using O_2_ saturated water or salt water (200 g L^–1^) solutions to monitor the reactive adsorption of phenol on the surface of the catalysts under oxygen-limited conditions (particularly for the highly concentrated NaCl solution). HPLC analysis of the phenol/O_2_/salt water solutions used in the ATR experiments highlighted that phenol did not undergo any partial oxidation (99.6% phenol remained according to HPLC).

To study the reactive adsorption of phenol, a flow of O_2_ saturated water/salt water (NaCl concentration: 200 g L^–1^) was introduced over the respective catalyst layers in the ATR flow cell. After stabilization, the cell was heated to 95 °C under the flow of O_2_-saturated water or salt water, denoted water/O_2_ and salt water/O_2_, respectively. Once the temperature of the cell was stable at 95 °C, 0.1 mol L^–1^ solution of phenol in water or salt water was passed over the catalyst layer, and ATR spectra were recorded (8 scans, resolution of 4 cm^–1^) before returning to a flow of water/O_2_ (salt water/O_2_) to assess the relative strength of adsorption of phenolic versus oxidized species. The background spectrum was that of the dry catalyst layer and the contribution of liquid water has been subtracted from all spectra shown.

## 3 Results and Discussion

### 3.1 Structural and Morphological Properties

The structural characteristics of the fresh MnCeO_*x*_ catalyst were determined using XRD, as presented in Figure S3a. Reflections at 2θ values of 28.6, 33.3, 47.5, and 56.5° indicate CeO_2_ with a fluorite-like structure [5], and reflections at 2θ values of 37.0, 38.2, 65.1, 66.2, 69.8, and 77.5° show the presence of MnO_*x*_ with the coexistence of multiple oxidation states.^27^ However, the low intensity of the MnO_*x*_ reflections prevent further phase identification.^28^ The broad CeO_2_ peaks could be attributed to a low crystallinity phase in the mixed oxides.^29^ N_2_ physisorption analysis of MnCeO_*x*_ shows a type IV isotherm (Figure S3b), which suggests a mesoporous structure of the MnCeO_*x*_ catalyst.^30^ In addition, the type H3 hysteresis loop in the isotherms indicates the presence of slit-like mesopores in MnCeO_*x*_.^29,31^ A narrow distribution of mesopores with a diameter of about 5.7 nm is shown in the inset of Figure S3b for MnCeO_*x*_. The BET surface area and the single point adsorption total volume at *p*/*p*^0^ = 0.99 of MnCeO_*x*_ were 191 m^2^ g^–1^ and 0.4 cm^3^ g^–1^, respectively. The morphological feature of MnCeO_*x*_ is shown in Figure S4a. SEM–EDS elemental maps of MnCeO_*x*_ (Figure S4b) show a homogeneous distribution of Mn and Ce phases across the MnCeO_*x*_ catalyst with a Mn/Ce atomic ratio of ∼1.3, which was comparable to the theoretical ratio of 1.5. This is in line with the results of X-ray fluorescence for the Mn/Ce atomic ratio, which was about 1.4. Transmission electron microscopy (TEM) images (as shown in Figure S5) present the microscopic features of the MnCeO_*x*_ catalyst, showing the random agglomerates of particles with irregular morphologies.

XPS analysis was performed to investigate the surface chemical state of Mn and Ce species in the MnCeO_*x*_ catalyst, as shown in Figure S6. The Ce XPS spectrum is typical of CeO_2_, or Ce^4+^, and results from the convolution of Ce 3d_5/2_ and Ce 3d_3/2_ spin–orbit coupling doublet signals, each of which comprises three further components due to various final-state 4f electron configurations.^32^ In the fresh catalyst, observation of fully oxidized ceria is expected since the catalyst has just been calcined in air. There is no evidence for the presence of a significant Ce^3+^ component, which, if present, would give rise to a prominent feature at ∼885 eV. Similarly, attenuation of the peak seen at ∼917 eV (which is only present in Ce^4+^) would be expected, whereas after fitting a Shirley background, we observe an intensity in this feature corresponding to ∼14% of the total Ce 3d features, which is characteristic of pure stoichiometric CeO_2_.^33^

Mn 2p XPS spectra have many multiplet-split components and are, therefore, challenging to fit reliably, but qualitative comparisons to reference spectra are still instructive.^34^ The absence of a shake-up satellite feature at ∼647 eV suggests that little, if any, Mn^2+^ is present. The absence of significant asymmetry of the Mn 2p_3/2_ spectra and the binding energy of 641.6 eV are more consistent with high-resolution literature data for Mn_2_O_3_ and manganite (MnOOH), both Mn^3+^, than that of MnO_2_ (Mn^4+^) samples, pointing to the initial sample being predominantly Mn^3+^. This is consistent with the proportions of different oxidation states reported in the literature for the MnO_*x*_–CeO_2_ catalysts.^35^ It should also be noted the presence of mixed Mn oxidation states is likely, based on the findings from the XRD that showed the presence of multiple MnO_*x*_ phases in the fresh catalyst,^28^ which can facilitate electron transfer and thus make an efficient catalyst for wet oxidation.^19^

The O 1s spectrum of the fresh MnCeO_*x*_ catalyst was deconvoluted to four peaks at 529.4, 532.0, 533.4, and 530.9 eV (Table S1). The spectra were deconvoluted using least-squares curve fitting with a mixture of Gaussian and Lorentzian functions on a Shirley-type background.^36^ The peaks at 529.4 and 530.9 eV were assigned to lattice and defective oxygen species of MnCeO_*x*_, respectively, with O 1s peaks at higher binding energies (532–533.5 eV) assigned to adsorbed OH/H_2_O.^34,35,37^

### 3.2 CWO over the MnCeO_*x*_ Catalyst

#### 3.2.1 CWO of Phenol in Water and Synthetic HISWW

**Figure 1:**
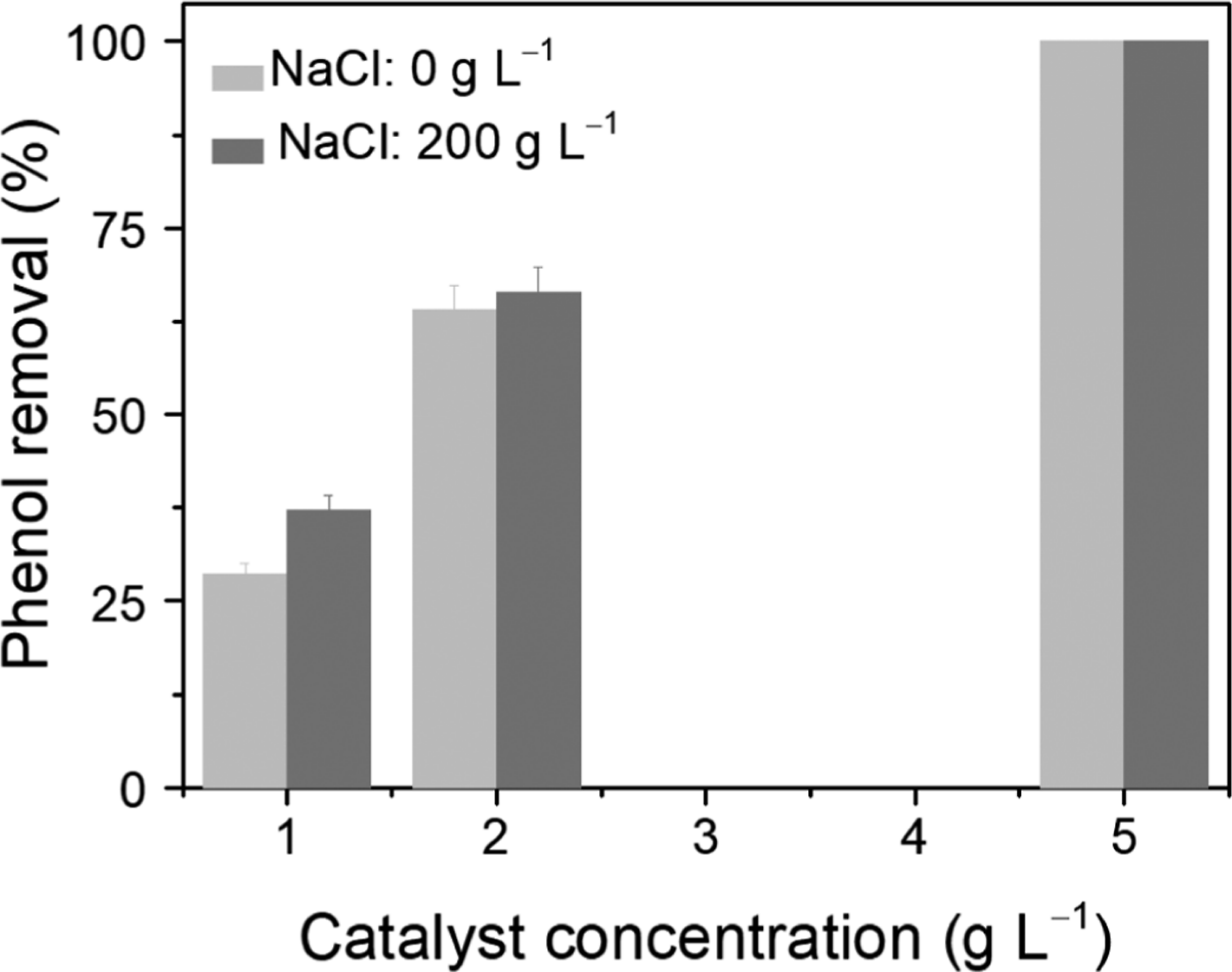
Phenol removals for reactions with different concentrations of the MnCeO_*x*_ catalyst of 1, 2, and 5 g L^–1^ (conditions: *C*_phenol_ = 1.0 g L^–1^, *T* = 110 °C, *P*_O_2__ = 0.5 MPa, and *t* = 2 h).

As presented in Figure 1, the CWO of phenol using the MnCeO_*x*_ catalyst in synthetic HISWW did not result in any detrimental effect on phenol removal, revealing that MnCeO_*x*_ exhibited good chloride resistance under these very high NaCl concentrations. At a catalyst concentration of 5.0 g L^–1^, complete removal of phenol and high TOC removal (∼91%) were achieved in the presence and absence of NaCl. The assessment of the carbon balance (see the Supporting Information) showed that the removal observed was mainly attributed to adsorption and formation of carbonaceous deposits (i.e., oxidized products/phenolic species) on the catalyst surface, and no mineralization was observed in the absence of NaCl. Interestingly, 10% phenol mineralization was observed in the salt water system, indicating that the presence of NaCl promoted phenol oxidation. At catalyst loadings of 1.0 and 2.0 g L^–1^, although no phenol mineralization was observed within 2 h of the reaction in the absence and presence of NaCl (see the carbon balance in the Supporting Information), phenol removal was enhanced in the salt water system; for example, the phenol removal increased to 39% in salt water from 28% for the pure water system after 2 h of the reaction with 1.0 g L^–1^ catalyst.

**Figure 2:**
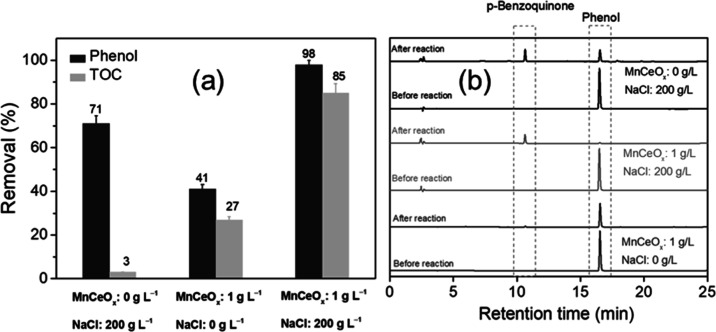
(a) Phenol and TOC removals in pure and salt water with different catalyst loadings (*C*_catalyst_ = 0.0 and 1.0 g L^–1^) and (b) HPLC spectra before and after these reactions (conditions: *C*_phenol_ = 1.0 g L^–1^, *T* = 110 °C, *P*_O_2__ = 0.5 MPa, and *t* = 24 h).

The enhancement in phenol removal in salt water at a MnCeO_*x*_ loading of 1.0 g L^–1^ was further evidenced at longer reactions times (Figure 2). In the absence of NaCl, the removal of phenol after 24 h was only 41%; however, almost complete removal of phenol (98%) was achieved in the presence of 200 g L^–1^ NaCl, as presented in Figure 2a. TOC removal was also enhanced, with 27 and 85% values observed in the absence and presence of salt, respectively. In addition, NaCl promoted phenol mineralization at this low catalyst concentration after 24 h (11% of the phenol feed introduced to the reactor was mineralized to CO_2_ in water, while in salt water, this increased to 51%, as presented in Table 1). This highlights the promoting effect of NaCl on CWO of phenol over MnCeO_*x*_.

**Table 1:** Carbon Balance in Phenol Oxidation (Reaction Conditions: *C*_phenol_ = 1.0 g L^–1^, *T* = 110 °C, *P*_O_2__ = 0.5 MPa, *C*_catalyst_ = 1.0 g L^–1^, and *t* = 24 h)^a^

	carbon (mg)			
	total carbon	liquid carbon	solid carbon	CO_2_
no NaCl	38.3	28.0	6.2	4.1
with NaCl	38.3	5.8	12.9	19.6

	carbon percentage (%)			
	total carbon	liquid carbon	solid carbon	CO_2_
no NaCl	100	73.1	16.2	10.7
with NaCl	100	15.1	33.7	51.2

a Errors are ±3%. No mineralization was observed in the homogeneous reaction.

CWO of phenol over MnCeO_*x*_ is clearly limited in terms of both partial oxidation of phenol and mineralization of intermediates, which was attributed to adsorption and the formation of carbonaceous deposits on the catalyst surface. These surface species limit the availability of sites for surface hydrocarbon oxidation reactions as well as the reoxidation of the catalyst surface. Surface oxidation of adsorbed phenol/intermediates is proposed as the rate-determining step in the CWO of phenol,^21^ and the activity of the catalyst for phenol removal is clearly related to the availability of active sites. Higher phenol removal was observed at a high catalyst-to-substrate ratio (Figure 1), while reduced phenol removal was noted in reactions with higher initial phenol concentrations (Figure S7). Therefore, the enhanced CWO of phenol in the presence of NaCl might be closely related to an effect of NaCl on the catalyst surface.

**Figure 3:**
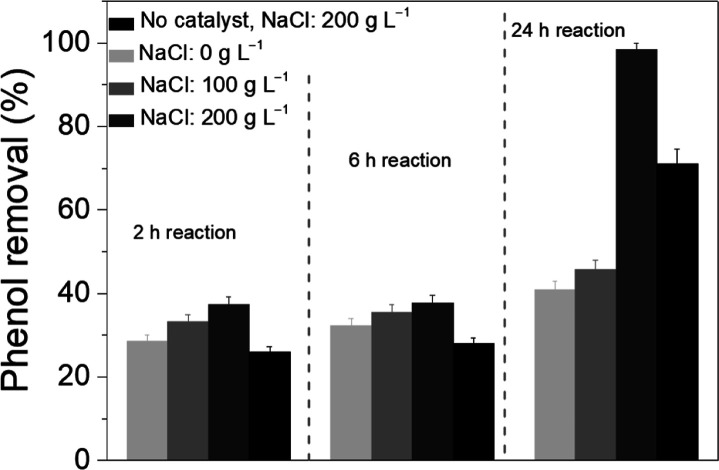
CWO over the MnCeO_*x*_ catalyst with the presence of NaCl (conditions: *C*_phenol_ = 1.0 g L^–1^, *C*_catalyst_ = 1.0 g L^–1^, *T* = 110 °C, and *P*_O_2__ = 0.5 MPa).

To clarify how NaCl promoted CWO of phenol in HISWW, phenol removal was measured in the absence and presence of MnCeO_*x*_, as shown in Figure 3. In the absence of MnCeO_*x*_, phenol removal was 71% after 24 h in salt water; however, the TOC before and after the reaction remained similar, with only a ∼3% decrease observed (Figure 2a). Analysis of the liquid phase showed that the presence of NaCl promoted a homogeneous reaction with the production of partial oxidation intermediates such as *p*-benzoquinone (0.53 mg/35.5 mg converted phenol, see Figure 2b) and short-chain carboxylic acids (e.g., fumaric acid, maleic acid, and oxalic acid, see Figure S8). In contrast, with no NaCl present, there was very little contribution from the homogeneous reaction, with only 2.8% phenol removal after a 24 h reaction being observed in the absence of MnCeO_*x*_. Complete oxidation of organics from the aqueous solution was not achievable without the catalyst. In the presence of MnCeO_*x*_, the increase in phenol removal for the reaction after 24 h in salt water could contain a contribution from the homogeneous partial oxidation reaction to benzoquinone and short-chain carboxylic acids, which could adsorb on the catalyst and undergo surface oxidation reactions (pathway to mineralization). However, as phenol adsorption on the catalyst occurs rapidly under reaction conditions, formation of carbonaceous deposits would be expected to dominate over the contribution of the homogeneous reaction. Indeed, concentrations of short-chain acids in the liquid phase after 24 h of the reaction in salt water with and without a catalyst were comparable (Figure S8), while the TOC conversion for these reactions were 85 and 3% with and without the catalyst, respectively. This highlighted the importance of the catalyst surface for the reaction and suggested that salt influenced the surface adsorption/oxidation of phenol to provide enhanced mineralization.

The promoting effect of NaCl on CWO of phenol was also observed over MnO_*x*_ and CeO_2_ in HISWW, as presented in Figure S9. The mixed oxide showed significantly higher activity than single oxides due to the enhanced redox ability of the mixed oxide, and the different catalytic performances of the single and mixed oxide catalysts in the presence of NaCl also indicated the important role played by the catalysts in the promoting effect of NaCl on CWO of phenol. Previous research regarding the effect of concentrated NaCl on CWO of oxalic acid over MnCeO_*x*_ has indicated that the presence of NaCl hindered oxalic acid conversion due to the salting-out effect, increasing the oxalic acid surface coverage with the formation of strongly adsorbed intermediates on the catalyst surface, slowing oxalic acid mineralization.^38^ In order to probe the effect of NaCl on CWO of phenol, in situ ATR-IR was employed to investigate the surface adsorption/oxidation of phenol over MnCeO_*x*_ (and single oxides) in the absence and presence of NaCl.

### 3.3 In Situ ATR-IR Study of CWO of Phenol over MnCeO_*x*_ in Water and Salt Water

**Figure 4:**
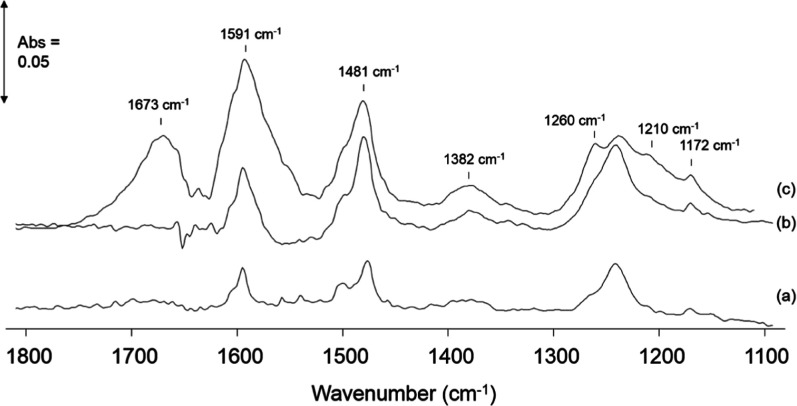
ATR-IR spectra of 0.1 M phenol/water/O_2_ over (a) ZnSe at RT (comparable spectrum over ZnSe at 95 °C), (b) MnCeO_*x*_ at RT, and (c) MnCeO_*x*_ at 95 °C. All spectra recorded after 15 min exposure to the phenol/water/O_2_ solution. Bands due to water have been subtracted.

The ATR-IR spectrum of phenol in water over the ZnSe crystal is shown in Figure 4, where bands at 1595 cm^–1^ (with a shoulder at 1605 cm^–1^), 1505, and 1477 cm^–1^ were assigned to the ν(C=C) ring vibrations and the band at 1172 cm^–1^ was assigned to the phenyl β(C–H) deformations. The 1387 cm^–1^ band was assigned to δ(OH) vibration with the ν(CO) stretching vibration observed at 1242 cm^–1^. These assignments correspond to those for phenol in aqueous solution as reported by Palmisano et al.^39^ The adsorption of phenol has been reported to occur molecularly on SiO_2_ through H-bonding interactions,^40^ while dissociative adsorption with the formation of phenolate species dominates over CeO_2_,^41^ Al_2_O_3_,^40^ and TiO_2_ surfaces.^42^ Dissociative adsorption of phenol on these metal oxides is characterized by the loss of bands corresponding to the phenol OH deformation (∼1380 cm^–1^) and a shift of the ν(C–O) vibration to higher wavenumbers (∼1270–1290 cm^–1^), indicating a strengthening (and shortening) of the C–O bond in the phenolate upon adsorption on the catalyst. The ATR-IR spectra of the adsorption of phenol over the MnCeO_*x*_ catalyst under O_2_-saturated water at RT and 95 °C shows that little adsorption of phenol occurred at RT, while reactive adsorption forming phenolates occurred at 95 °C.

**Figure 5:**
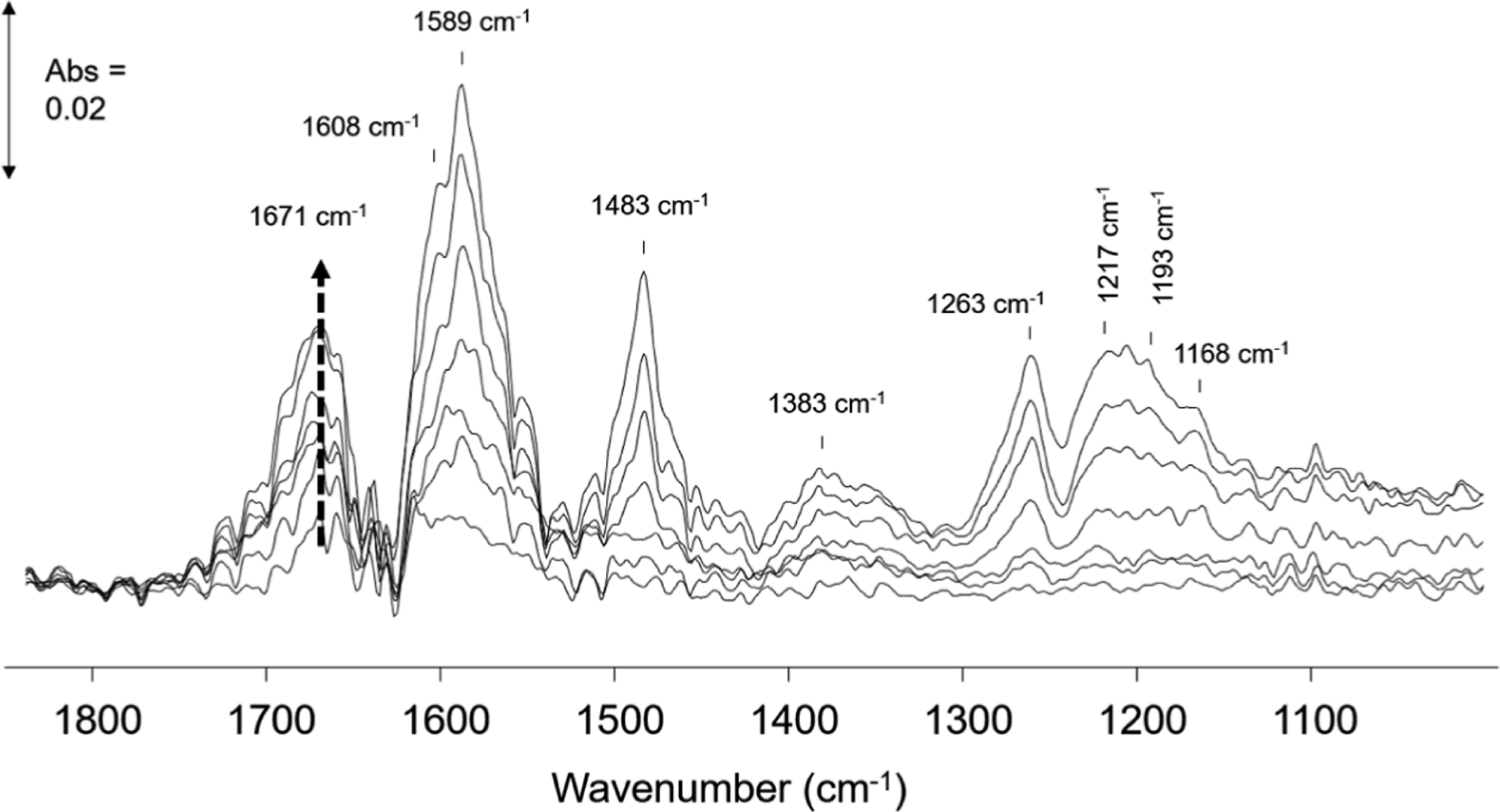
ATR-IR spectra of 0.1 M phenol over MnCeO_*x*_ in water/O_2_ at 95 °C under increasing exposure time to the phenol solution. Bands due to water and liquid-phase phenol have been subtracted.

The spectra in Figure 5 were recorded under a flow of phenol/O_2_/water, and, in the initial 2 min of exposure of MnCeO_*x*_ to the phenol solution, bands (1600–1700 cm^–1^ with the main band at 1671 cm^–1^) due to adsorbed partial oxidation products were observed to form. Bands in the 1600–1700 cm^–1^ region have also been observed on CeO_2_ following low-temperature ozonolysis of phenol^43^ and also in spectra over vanadia–titania catalysts during the oxidation of benzene, wherein these bands were assigned to partial oxidation intermediates and adsorbed quinones (∼1680 cm^–1^ to *o*-quinone and ∼1660 cm^–1^ to *p*-quinone), respectively.^44^ In addition, bands in the 1700 cm^–1^ region have been reported in the photodecomposition of phenol over TiO_2_ and assigned to the oxidation of phenol to carboxylic acids such as oxalic acid (bands in the 1715 and 1690 cm^–1^ regions are attributed to oxalate species chemisorbed on TiO_2_).^42^ The formation of the bands at 1600–1700 cm^–1^ indicate the oxidation of phenolates to quinones/carboxylic acids (ring opened) over MnCeO_*x*_ in the conditions used in this ATR study.

The initial interaction of phenol with the catalyst surface resulted in the oxidation of phenol over the MnCeO_*x*_ catalyst. As the exposure time to the phenol solution increased, additional bands due to phenolate species were observed to form. Characteristic bands for phenolates adsorbed on Ce and Mn sites in MnCeO_*x*_ were identified with the ν(CO) of Ce phenolates assigned to the band at 1263 cm^–1^, while on Mn sites, this band was shifted to 1217 cm^–1^ (band assignments are detailed in the Supporting Information).

The ν(CO) bands of phenolates on MnCeO_*x*_ were at comparable positions in the mixed oxide to those of the respective single oxides (Figures S10 and S11), suggesting the intrinsic adsorption properties of the Ce and Mn sites in the mixed oxide catalyst. Interestingly, in addition to comparable band positions, the relative strength of adsorption of the partial oxidation products and phenolate species adsorbed on MnCeO_*x*_ sites were comparable to that observed for the single oxides (see the Supporting Information and Figures S12 and S13). Phenolates adsorbed on Ce sites in MnCeO_*x*_ showed weaker adsorption with a reduction in band intensity under a flow of water/O_2_ at 95 °C (Figure S14). As these Ce phenolate bands decreased, bands due to the oxidized products increased, highlighting the conversion of phenolates to oxidized products on Ce sites in MnCeO_*x*_. Bands due to the Mn phenolates and Ce-oxidized products were retained on MnCeO_*x*_ after the water/O_2_ flow at 95 °C.

These ATR-IR experiments were performed in the regime of reactive adsorption and formation of carbon deposits on the catalyst surface as opposed to conditions of complete mineralization (130–160 °C and oxygen partial pressures of 1–2 MPa).^23^ The bands due to partially oxidized species over CeO_2_ and MnCeO_*x*_ (relatively weak bands observed for MnO_*x*_) being at comparable wavenumbers and formed under the same reaction conditions suggest that in the mixed oxide catalyst, quinones/acids were predominantly adsorbed on Ce sites under these conditions (95 °C, O_2_-saturated water). The rate of formation of these bands due to oxidized species was slower on CeO_2_ than over the MnCeO_*x*_ catalyst and provides evidence in support of the enhanced oxidation ability (redox properties) of the mixed oxide catalyst compared to that of CeO_2_ alone, which was the least active of the catalysts tested (Figure S9). The retention of partially oxidized products on Ce sites and phenolics on Mn sites of MnCeO_*x*_ correlates with the study of Hamoudi et al., where using XPS and NMR, they showed carbon deposits to contain both aromatic and COOH functionalities;^24^ however, in situ ATR spectroscopy has allowed the identification of the nature of the adsorbed species (phenolate vs partial oxidation products) as well as the adsorption site (Ce vs Mn) and relative strength of adsorption, indicating the nature of the strongly bound carbonaceous deposits retained on the catalyst to be linked to the products of partial oxidation of surface-bound phenolates.

**Figure 6:**
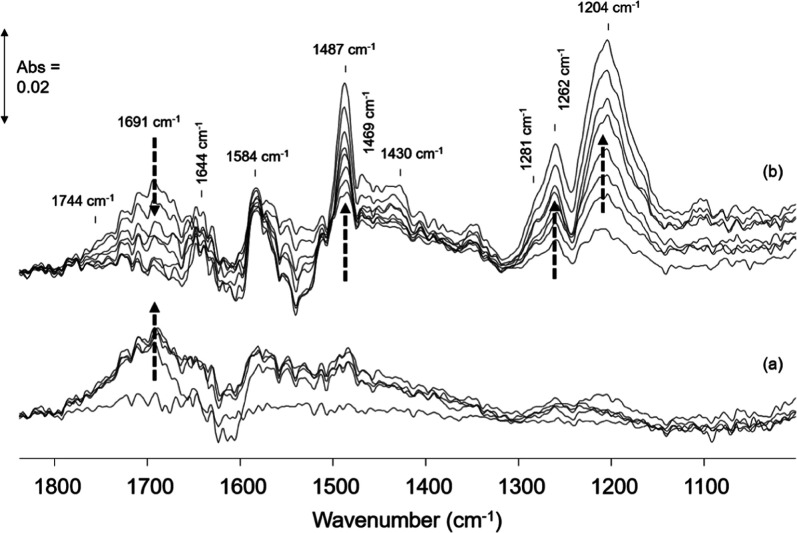
ATR-IR spectra of MnCeO_*x*_: (a) 0–2 min of exposure and (b) 2–15 min of exposure to 0.1 M phenol in salt water/O_2_ at 95 °C. Bands due to water and liquid-phase phenol have been subtracted.

Herein, CWO of phenol in high-ionic-strength water was shown to enhance the mineralization of the carbon deposits formed on MnCeO_*x*_ from the reactive adsorption of phenol. ATR-IR spectra of the carbon deposits formed on MnCeO_*x*_ in O_2_-saturated high-ionic-strength water are shown in Figure 6. As in water, the initial interaction of phenol with MnCeO_*x*_ resulted in oxidation with the formation of adsorbed quinone/carboxylic acid species. The formation of partially oxidized products in salt water and water, the former having significantly lower dissolved O_2_ content,^45^ indicates the role of lattice oxygen in MnCeO_*x*_ in the oxidation of adsorbed phenol. Phenol adsorption in salt water showed a shift in the main band in the region of partially oxidized products from 1671 cm^–1^ in water to 1691 cm^–1^ (with a shoulder at 1744 cm^–1^) in salt water, which suggests the formation of new oxidation products or a change in the environment of the quinones/acids when adsorbed on MnCeO_*x*_ in the presence of NaCl. Indeed, the same adsorbed species forming in water and salt water (along with the comparable liquid-phase products identified in the reaction) suggest the same reaction mechanism occurring in water and salt water with the oxidation rate/extent of mineralization enhanced in the presence of concentrated NaCl.

Interestingly, the intensity of the bands due to oxidized products increased under the phenol/salt water flow, reaching a maximum intensity after ∼2 min before decreasing as bands due to adsorbed phenolates (on Ce and Mn sites) began to form (Figure 6). The fact that adsorbed quinones/acid products are retained on the catalyst in water but are observed to undergo further reaction in salt water is in excellent agreement with the activity data, where enhanced mineralization was observed for reactions in salt water.

It is also interesting to note that the relative intensities of the ν(CO) bands of Ce and Mn phenolates over MnCeO_*x*_ were of comparable values in water (Figure 5), while in salt water (Figure 6), the ν(CO) bands of the Mn phenolates were relatively more intense than those of the Ce phenolates. This suggests that the modification of the carbon deposit adsorption/surface coverage on Ce and Mn sites of MnCeO_*x*_ was influenced by the presence of NaCl.

The difference in the observed adsorption of phenol between water and salt water can be attributed to an effect of pre-exposing the catalyst to salt water while heating to 95 °C, which altered the phenol adsorption mode and surface coverage. Comparison of the reactive adsorption of phenol over the single oxide catalysts in salt water showed that NaCl dramatically hindered the reactive adsorption of phenol on Ce sites (Figure S12), while for MnO_*x*_, the reactive adsorption of phenol was enhanced in salt water (Figure S13).This differing adsorption behavior of phenol in salt water could be the result of electrostatic interactions due to the differing surface charges of the single oxides (zero point charge, ZPC of CeO_2_ = 8^46^ and ZPC of MnO_2_ = 3^47^), which in salt water influences the dissociative adsorption of phenol.

**Scheme 1:**
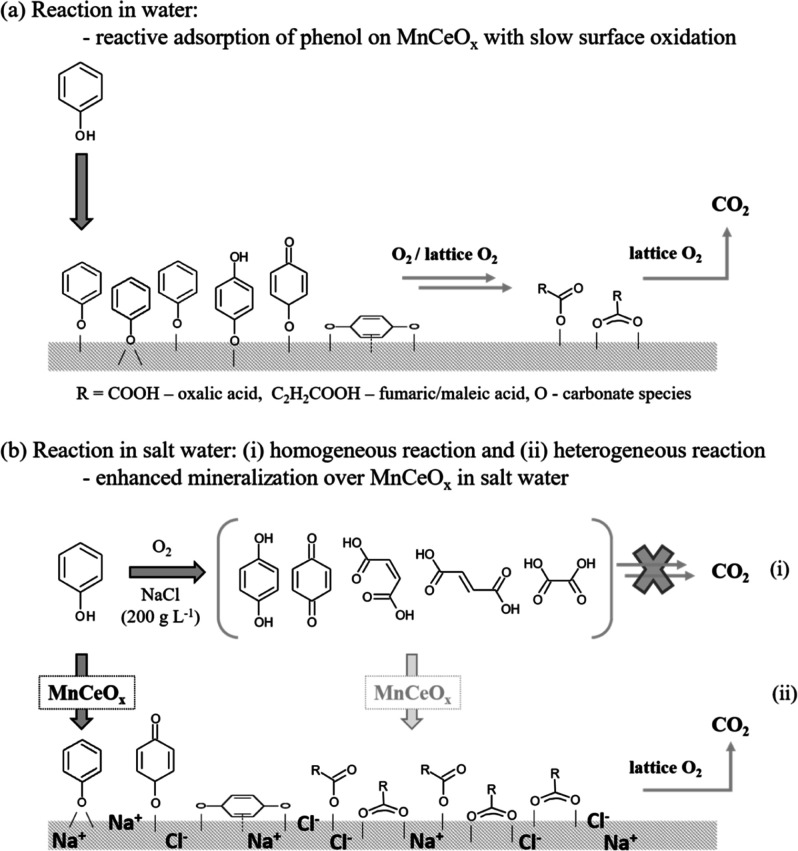
Schematic of CWO Reaction Pathways in Water and Salt Water

The ATR experiments show the adsorption of phenol and partial oxidation products on MnCeO_*x*_ in water and salt water equating to the carbonaceous deposits formed on the catalyst, which undergo slow surface oxidation on the pathway to mineralization. The differing relative intensity of phenolate/partial oxidation products adsorbed on the catalyst in salt water point to the enhanced mineralization of the carbon deposits in the presence of salt, which is attributed to the blocking of sites for the reactive adsorption of phenol (and/or electrostatic interactions altering the phenol adsorption), resulting in the reduction of the carbon deposit coverage over the catalyst. Ce sites, which have been shown to oxidize adsorbed phenol to strongly adsorbed quinone/carboxylic acid intermediates, are also the sites that show the largest reduction in phenol adsorption in salt water. The reduced coverage of the catalyst surface with strongly bound carbon deposits (oxidized quinone/acid species on Ce sites) would aid the activation of O_2_ at the catalyst surface/replenishment of lattice oxygen, in line with the proposed Langmuir–Hinshelwood mechanism for the CWO of phenol^19^ (and the Mars–van Krevelen mechanism for oxalic acid, an intermediate in the oxidation of phenol^38^), as shown in Scheme 1.

### 3.4 Deactivation and Regeneration

**Figure 7:**
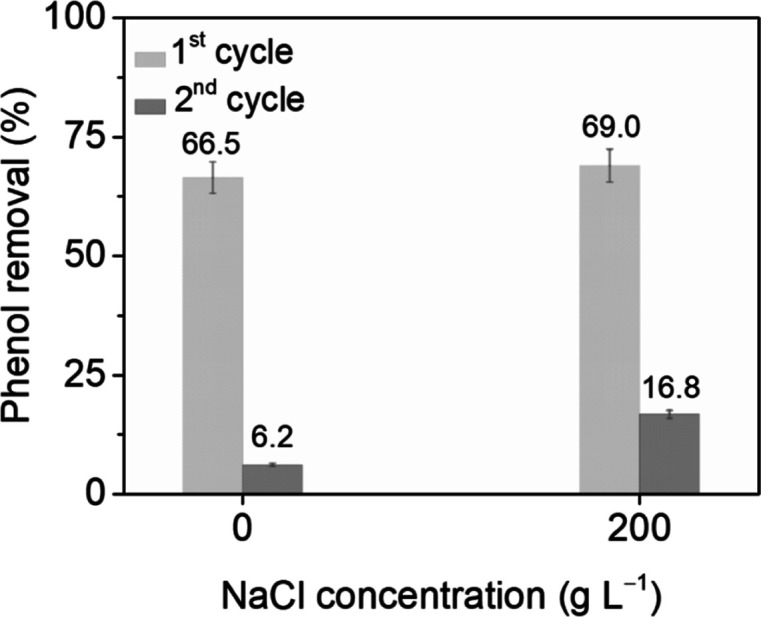
Stability evaluation of the MnCeO_*x*_ catalyst in CWO of phenol with pure and salt water (conditions: *C*_phenol_ = 1.0 g L^–1^, *P*_O_2__ = 0.5 MPa, *C*_catalyst_ = 2.0 g L^–1^, and *t* = 2 h).

To date, the deactivation of MnCeO_*x*_ catalysts during CWO of phenol has been proposed to be related to the build-up of carbon deposits and the loss of reversibility of the surface redox cycle.^20^ As comparable phenol removals were observed in the absence and presence of NaCl at a catalyst concentration of 2.0 g L^–1^, the deactivation of the catalyst at this concentration was assessed by recycling the catalyst to probe whether the carbon deposit formation was influenced by the presence of NaCl, as shown in Figure 7. Under these conditions of adsorption (and no mineralization), although deactivation was observed on recycling the catalyst under the same conditions in both water and salt water, for the catalysis in pure water, the phenol conversion decreased from 67% (in the first test) to 6% (in the second test), while a slightly improved performance was observed in the presence of 200 g L^–1^ NaCl, where conversion in the recycle reaction was 17%, which suggested a differing nature of the carbonaceous deposits after the reaction in salt water or a different coverage of the catalyst surface with some active sites still available for phenol adsorption.

XRD characterization of the fresh and used MnCeO_*x*_ catalysts showed comparable diffraction patterns (as shown in Figure S15). However, for the used catalyst from the salt water system, a characteristic peak of NaCl was clearly identified. N_2_ physisorption analysis of the fresh and used MnCeO_*x*_ catalysts (from the systems with pure and salt water) showed an ∼53% decrease in the pore volume from 0.4 cm^3^ g^–1^ for the fresh catalyst to 0.2 cm^3^ g^–1^ for the used catalyst in the absence and presence of salt. Both these observations are entirely consistent with deactivation by organic or carbonaceous deposits at the surface, blocking pores but not altering the bulk of the material.

XPS analysis of the used MnCeO_*x*_ catalyst is shown in Figure S16. The intensity of the spectra is considerably reduced compared with that of the fresh catalyst because of the presence of carbonaceous or organic material, as expected from the ATR result. Nevertheless, both the Mn 2p and Ce 3d spectra obtained after the reaction are very similar to those obtained prior to the reaction. For the Mn 2p spectra, there is a very slight increase in the binding energy of the center of the 2p_3/2_ signals, which may point to a slight increase in oxidation, but this is <0.2 eV (and the typical difference is expected to be ∼0.5 eV between Mn^3+^ and Mn^4+^).^34^ It must also be noted that the significant attenuation in the signal due to the carbonaceous or organic overlayer is also changing the XPS probing depth, which could be a source of any small changes seen. For the Ce 3d spectra, the feature at ∼917 eV remains ∼14% of the overall integrated Ce 3d signal, indicating that the sample is still overwhelmingly Ce^4+^. Overall, there is little change in the oxidation state of the metals in the oxide catalyst between the pre- and post-reaction samples.

**Figure 8:**
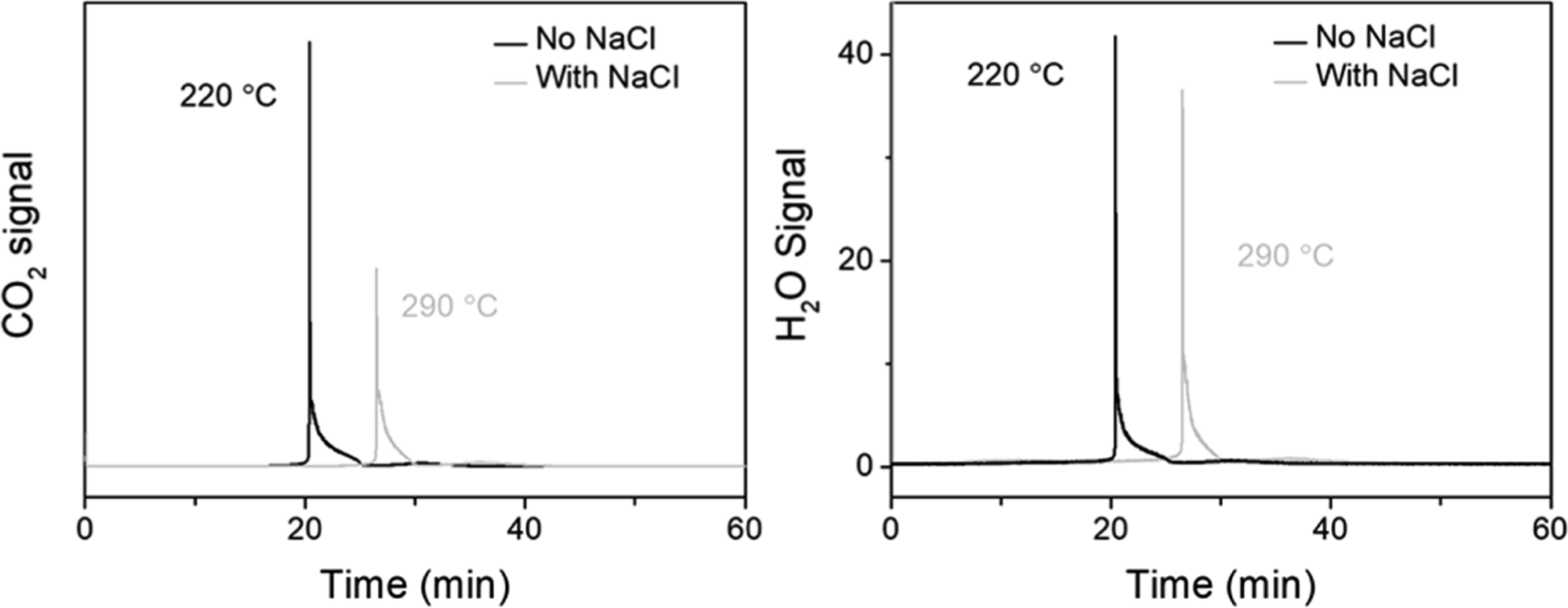
TPO-MS profiles of used MnCeO_*x*_ catalysts (conditions: *C*_phenol_ = 1.0 g L^–1^, *P*_O_2__ = 0.5 MPa, *C*_catalyst_ = 2.0 g L^–1^, and *t* = 2 h).

TPO-MS was carried out to study the formation of carbonaceous deposits on the used catalysts, and the evolution of CO_2_ and H_2_O is shown in Figure 8. O_2_ consumption of the two used catalysts during TPO correlated with the MS profiles for the formation of CO_2_ and H_2_O (Figure S17). The area under the peaks in the MS signals during the TPO measurements of the used catalysts in relation to the amount of oxidizable carbonaceous/organic deposits is presented in Table S2. The amount of CO_2_ produced from the used MnCeO_*x*_ catalyst in the salt water system was ∼8% lower than that from the pure water system (the amount of H_2_O produced from the used MnCeO_*x*_ catalyst in the salt water system was ∼7% lower than that from the pure water system), which suggested that the catalysis in salt water prevented the formation of carbonaceous species on the catalyst to some degree. In addition, the temperature range over which deposits’ oxidation occurred varied for the differently treated used catalysts. The catalyst following the reaction in salt water required a higher temperature (∼300 °C vs ∼200 °C) to oxidize the adsorbed carbon deposits than the catalyst from the pure water reaction. The nature of the carbon deposits did not change with the reaction time in salt water, always showing a peak at ∼300 °C. However, the temperature required to oxidize the adsorbed carbon deposits on the catalyst used in pure water shifted from 220 °C after 2 h of the reaction (when there was no mineralization) to 300 °C after 24 h of the reaction (∼10% mineralization was observed), see Figure S18 and Table 1. The nature of the carbonaceous deposits retained on the catalyst in water changing with reaction time indicates that mineralization was driven by the oxidation of species adsorbed on the catalyst surface, which is the rate-limiting step in CWO of phenol, and that the presence of NaCl promoted mineralization.

**Figure 9:**
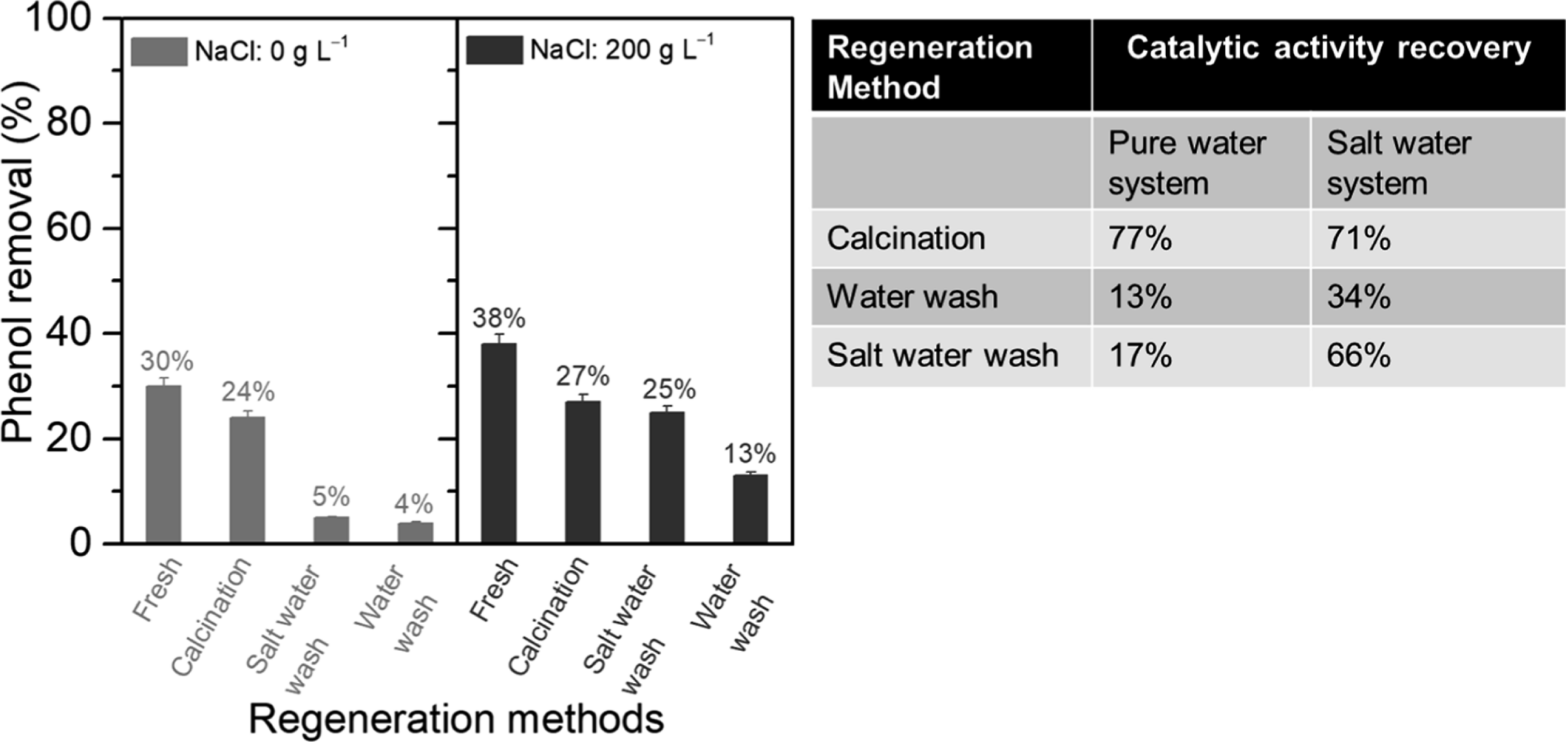
Phenol removals by the fresh and regenerated MnCeO_*x*_ catalysts (conditions: *C*_phenol_ = 1.0 g L^–1^, *P*_O_2__ = 0.5 MPa, *C*_catalyst_ = 1.0 g L^–1^, and *t* = 2 h).

Although catalyst deactivation was observed, the used MnCeO_*x*_ catalyst after the catalysis in pure water was able to be regenerated via calcination at 300 °C, which could remove the deposited carbonaceous species on the catalyst surface. Herein, calcination was used as a reference regeneration method for reactions with 1 g L^–1^ phenol and 1 g L^–1^ catalyst for only 2 h, where the high extent of reactive adsorption occurred as opposed to mineralization to CO_2_. As shown in Figure 9, the catalytic reaction in pure water resulted in phenol conversions of ∼30 and 23% after a 2 h reaction over the fresh and regenerated (via calcination) catalysts, respectively. The catalytic activity recovery was calculated according to eq S1, and for the used catalyst from the pure water system, the catalytic activity recovery was about 77% after calcination. Only a small amount of Mn leaching (<1%) was observed during the reaction, in line with the insignificant leaching of Mn and Ce reported by Hamoudi et al.,^24^ which could contribute to the small reduction in the catalytic activity observed upon recycling the regenerated catalysts (Table S3). It should be noted that no significant change was found in the crystal structure or oxidation state of the regenerated MnCeO_*x*_ catalyst, as shown in Figures S15 and S16. For the catalysis in salt water, the catalytic activity recovery via calcination was 71%.

A milder regeneration strategy was also tested using water and salt water washes under reaction conditions. The catalytic activity recovery by pure water wash under O_2_ was ∼13%, while using the salt water wash under O_2_, 66% catalytic activity was recovered, indicating that a salt water wash under the reaction condition could be another efficient way to regenerate the used catalyst and suggests that NaCl has altered the carbonaceous deposits on the surface of MnCeO_*x*_ in the first reaction to be more easily mineralized, aiding the recovered activity under mild regeneration conditions in the subsequent reaction. Indeed, the salt water wash was unable to regenerate the used catalyst after an initial reaction in pure water, which agrees with the reduced coverage/changed nature of the adsorbed deposits on MnCeO_*x*_ following reactions in the presence of NaCl. The ability to remove the adsorbed carbonaceous deposits and regenerate 71% of the catalyst activity via calcination at 300 °C and 66% via a salt water wash (after the reaction in salt water) for the catalyst was excellent and would indicate that the regeneration of the catalyst following reactions where mineralization was enhanced (higher catalyst: substrate ratio, addition of NaCl for a longer reaction time, and higher temperature/O_2_ partial pressure) would bring additional benefits for the regeneration activity of MnCeO_*x*_.

## 4 Conclusions

CWO of phenol over the MnCeO_*x*_ catalyst in the absence and presence of high NaCl concentration (mimicking the high salinity of HISWW) was studied, and complete removal of phenol could be achieved at 110 °C in 2 h with a catalyst loading of 5.0 g L^–1^. A significant promoting effect of NaCl on CWO of phenol was observed. When the NaCl concentration was 200 g L^–1^, phenol and TOC conversions were 98 and 85% (adsorption on the catalyst and oxidation reactions), and mineralization under these conditions reached 51% after a 24 h reaction using MnCeO_*x*_ at a concentration of 1.0 g L^–1^. Under the same conditions in the absence of NaCl, only 11% mineralization was observed, and the phenol and TOC conversions were 41 and 27%, respectively. This is a striking result highlighting the excellent chloride resistance of MnCeO_*x*_, in contrast to that of widely reported supported metal catalysts, which are easily poisoned by halide ions. In situ ATR-IR spectra during the CWO of phenol over MnCeO_*x*_ showed that NaCl reduced the carbon deposit coverage, which could promote surface oxidation/O_2_ replenishment of the catalyst, resulting in enhanced mineralization. Carbonaceous deposits of differing natures were observed on the used catalysts in the presence and absence of NaCl, and the used catalyst in the salt water system was able to be efficiently regenerated via a salt water wash under the reaction conditions. The promoting effect of NaCl on phenol mineralization over the MnCeO_*x*_ catalyst was related to NaCl hindering the reactive adsorption of phenol and the carbon deposit coverage over the catalyst. As current industrial CWO processes predominantly utilize noble metal catalysts that would be unsuitable for HISWW, the high activity and excellent chloride resistance of MnCeO_*x*_ shows potential for practical application in the oxidation of pollutants in highly concentrated NaCl waste streams as well as oxidation of chlorinated pollutants.

## Supporting Information Available

Control experiments of phenol oxidation; catalyst characterizations; carbon balance; effect of initial phenol concentration; HPLC analysis; comparison of the CWO of phenol over CeO_2_, MnO_*x*_, and MnCeO_*x*_ in water and salt water; in situ ATR-IR study of CWO of phenol over CeO_2_ and MnO_*x*_; and recycle reactions and regeneration of used MnCeO_*x*_ (PDF)
