# Supplementary material for: High-Ionic-Strength Wastewater Treatment via Catalytic Wet Oxidation over a MnCeOx Catalyst
Source: ACS Catal. 2022 Jun 13;12(13):7598–608. doi: 10.1021/acscatal.2c01952 (PMC9251724; doi:10.1021/acscatal.2c01952)
Supplement: Supplementary file 1 — cs2c01952_si_001.pdf [file cs2c01952_si_001.pdf]

High-Ionic-Strength Wastewater Treatment via Catalytic Wet Oxidation over $MnCeO_x$ Catalyst

Electronic Supplementary Information (ESI)

Xiaoxia Ou,[a,*] Helen Daly,[a,*] Xiaolei Fan,[a] Simon Beaumont,[b] Sarayute Chansai,[a] Arthur Garforth,[a] Shanshan Xu[a], Christopher Hardacre[a,*]

[a] *Department of Chemical Engineering, School of Engineering, The University of Manchester, Oxford Road, Manchester, M13 9PL United Kingdom*

[b] *Department of Chemistry, University of Durham, South Road, Durham, DH1 3LE, United Kingdom*

1. Control experiments of phenol oxidation

Blank experiments under N_2 and O_2 (at 110 °C and 0.5 MPa) were performed without the $MnCeO_x$ catalyst to investigate the effect of NaCl on the oxidation of phenol in the aqueous systems. **Fig. S1a** shows that in the absence of NaCl, phenol removal after 2 h of reaction was insignificant, with only 1.6% and 2.2% removal found under N_2 and O_2, respectively. This is as expected since wet oxidation of phenol is typically carried out at elevated temperatures (125–320 °C) and pressures (0.5–20 MPa) [1] in comparison with CWO. An increase in the system salinity (*i.e.* C_{NaCl} = 200 g L^{-1}) resulted in enhanced phenol removal for reactions under N_2 (6.1%±0.2%) and O_2 (~26.0%) (**Fig. S1a**). A promoting effect of NaCl on homogeneous oxidation of alcohols has been reported [2], and NaCl is known to decrease the solubility of reactants in aqueous solution (acting as a salting-out agent), which can make reactant molecules more hydrophobic, and hence, forcing reactant molecules into contact to accelerate the reaction [3]. In this work, the increased phenol removal with the addition of NaCl under N_2/O_2 in the absence of MnCeOx could be from the homogeneous reaction [2-4].

Little adsorption of phenol was observed over $MnCeO_x$ at RT under N_2 ($C_{catalyst}$ = 5.0 g L^{-1}) for the CWO of phenol (**Fig. S1b**). However, at 110 °C under N_2, phenol removal was found to be 29.6% over $MnCeO_x$, which could be attributed to the reactive adsorption of phenol or reaction between phenol and lattice oxygen of the catalyst (the intimate interaction between Mn and Ce can improve the oxygen

* Corresponding author e-mail address: xiaoxia.ou@manchester.ac.uk (X. Ou)

* Corresponding author e-mail address: helen.daly@manchester.ac.uk (H. Daly)

* Corresponding author e-mail address: c.hardacre@manchester.ac.uk (C. Hardacre)

storage capacity of $MnCeO_x$ which is linked to the high activity of these catalysts) [5]. Intermediate products (*e.g.* p-benzoquinone) were observed in the liquid phase which suggested a degree of phenol oxidation occurred under these conditions. The presence of NaCl in the CWO over $MnCeO_x$ under N_2 promoted phenol removal from 29.6% to 40.5%. Such enhancement of catalytic performance could be attributed to the conversion of phenol to intermediate oxidation products and/or the salting-out effect causing enhanced adsorption of phenol (and/or intermediates) onto the catalyst surface [6].

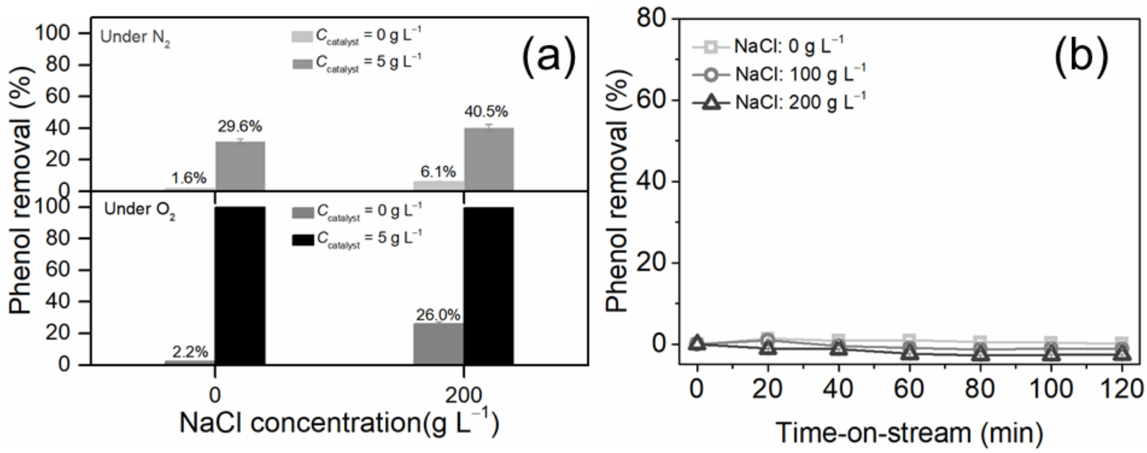

Fig. S1. (a) Comparative blank and CWO experiments under N_2 and O_2 (conditions: C_{phenol} = 1.0 g L^{-1}, T = 110 °C, P = 0.5 MPa, t = 2 h) and (b) Phenol removal over $MnCeO_x$ catalyst (5.0 g L^{-1}) under N_2 atmosphere at room temperature.

2. Catalyst characterisations

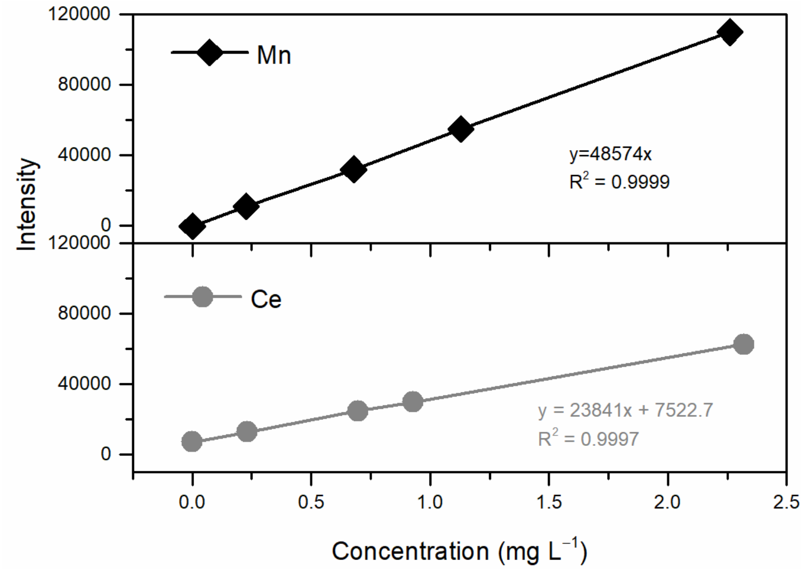

Fig. S2. ICP-OES calibration curves for manganese (top) and cerium (bottom) ions.

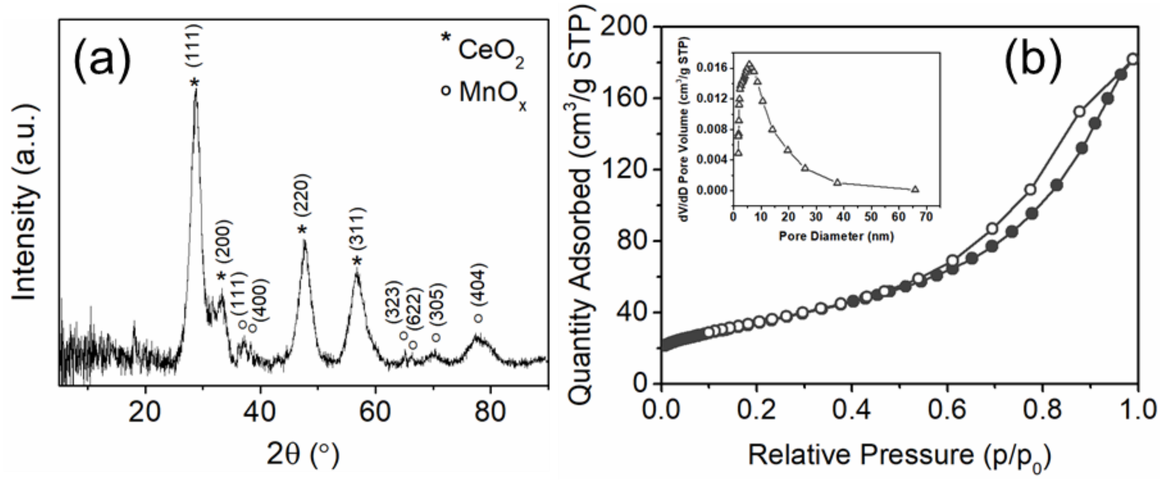

Fig. S3. (a) XRD pattern and (b) N_2 adsorption-desorption isotherms (inset: pore size distribution of $MnCeO_x$ derived from the adsorption branch of the isotherm) of the as-prepared (fresh) $MnCeO_x$ catalyst.

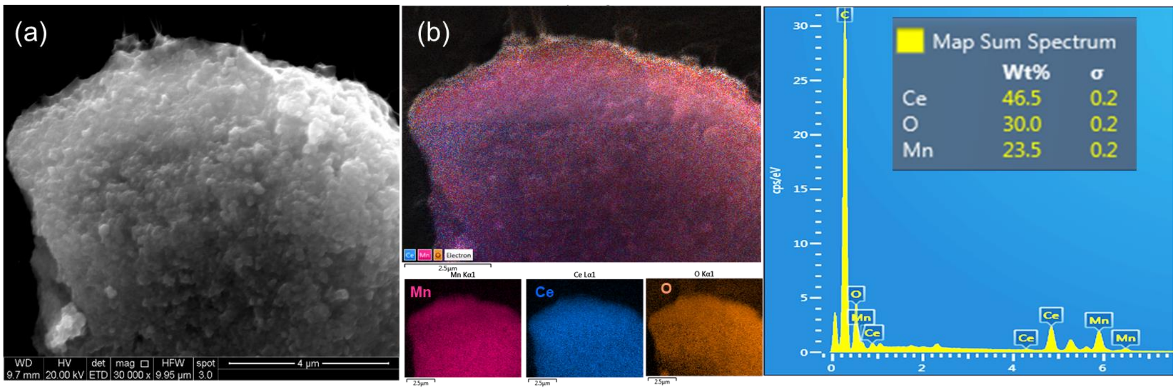

Fig. S4. (a) SEM micrograph of $MnCeO_x$; (b) EDS maps of $MnCeO_x$ with elemental information on Mn, Ce and O.

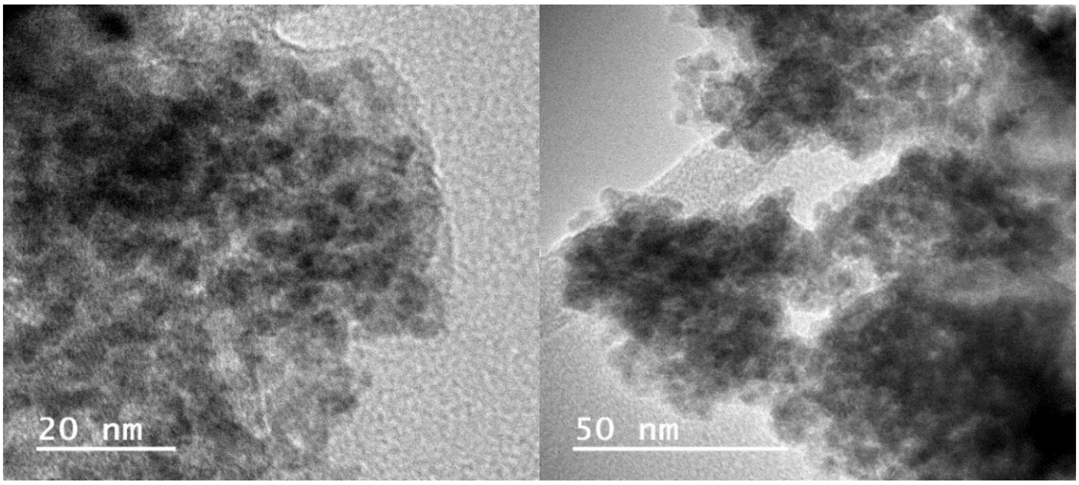

Fig. S5. TEM micrographs of the $MnCeO_x$ catalyst.

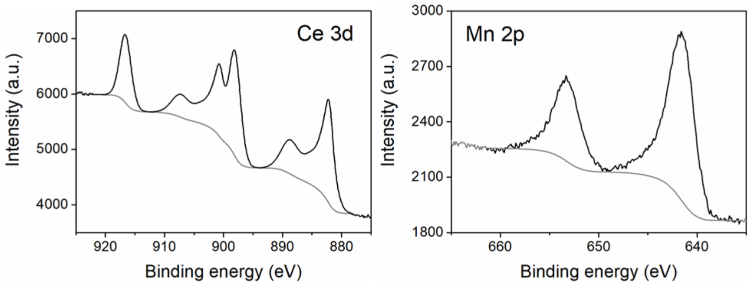

Fig. S6. XPS spectra of Ce 3d and Mn 2p of fresh $MnCeO_x$.

Table S1

O 1s XPS peak analysis of fresh $MnCeO_x$ catalyst.

	Peak 1		Peak 2		Peak 3		Peak 4	
O	eV	area/%	eV	area/%	eV	area/%	eV	area/%
Fresh	529.4	33	532.0	33	533.4	28	530.9	7

3. Carbon balance

The TOC removal in the pure and salt water systems after 2 h of reaction at 110 °C and 0.5 MPa O_2 were 62.8% and 56.1% when the concentrations of $MnCeO_x$ catalysts were 2 g L^{-1}. The feeding of phenol at concentration of 1 g L^{-1} contained 0.53 mmol phenol equivalent in 50 ml reaction solution, whereas at the end of the reaction the TOC concentration was reduced to 0.20 and 0.23 mmol phenol equivalent. According to the TPO-MS data, the amounts of carbon deposits adsorbed on the surface of the used catalysts in the pure and salt water systems were 2.17 mmol and 1.94 mmol. Therefore, the total carbon in the pure and salt water systems (including in the aqueous solution and adsorbed on the catalyst surface) were 3.37 and 3.32 mmol, demonstrating the carbon balance reached 100% (with errors of 5%) in this study and no mineralisation was observed in both systems when the catalyst concentration was 2 g L^{-1} or below (after 2 h of reaction).

Increasing the catalyst concentration to 5 g L^{-1}, TOC removals were ~91% in both systems, namely 2.90 mmol organic carbon was removed from the aqueous solution with initial carbon concentration of 3.19 mmol in the feed in the absence and presence of NaCl. The carbonaceous deposit on the surface of $MnCeO_x$ catalysts were calculated from TPO-MS data of the spent catalysts in the pure water and salt water systems, which were 3.05 and 2.57 mmol, respectively. Herein, reaction in water resulted only in adsorption of the phenol while 0.33 mmol CO_2 (about 10% of the feed) was produced in the salt water system.

4. Effect of initial phenol concentration

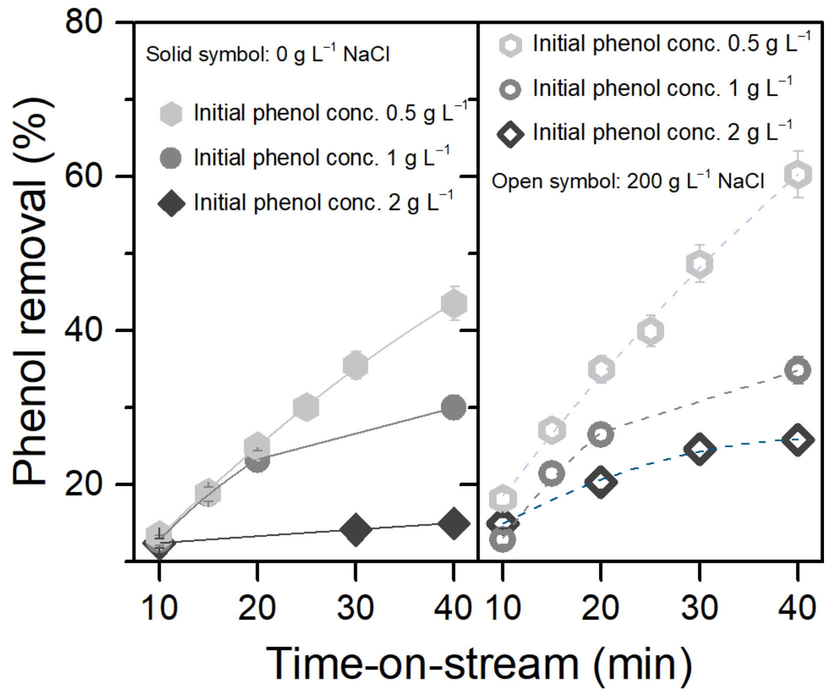

Fig. S7. Phenol removal for reactions with different initial phenol concentrations of 0.5, 1 and 2 g L^{-1} (conditions: 1.0 g L^{-1} $MnCeO_x$, P_{O2} = 0.5 MPa, T = 110 °C)

5. HPLC analysis

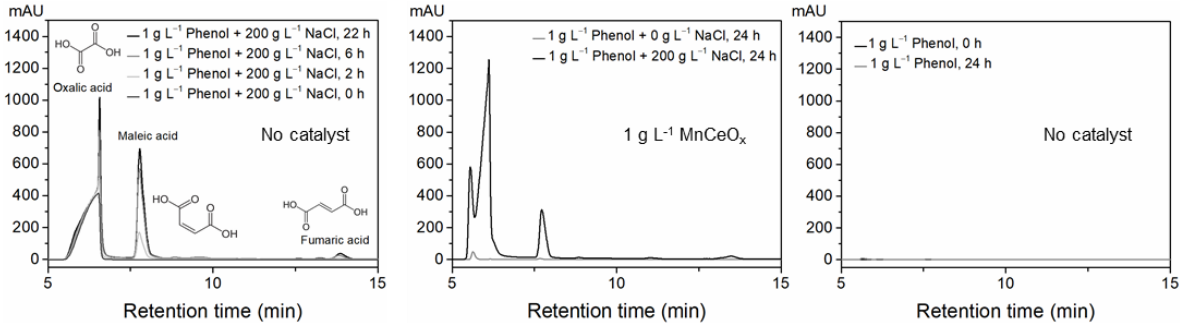

Fig. S8. HPLC spectra of small organic acids in different treated samples (conditions: P_{O2} = 0.5 MPa, T = 110 °C).

6. Comparison of the CWO of phenol over CeO_2, MnO_x and $MnCeO_x$ in water and salt water

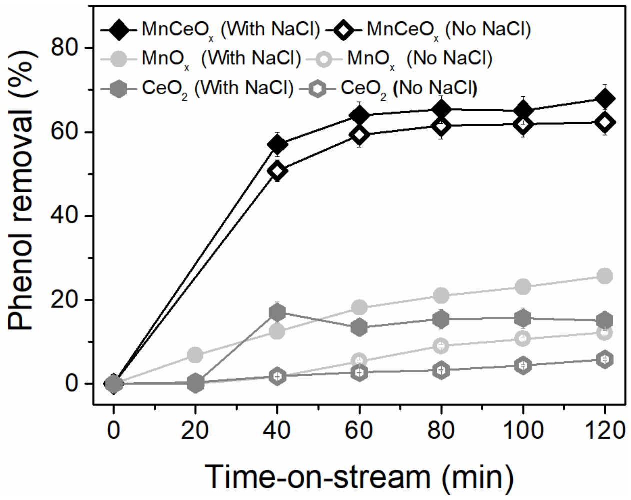

Fig. S9. Phenol removals with ToS over MnO_x, $MnCeO_x$ and CeO_2 (conditions: C_{phenol} = 1 g L^{-1}, T = 110 °C, P_{O2} = 0.5 MPa, 2 g L^{-1} catalyst).

7. In situ ATR-IR study of CWO of phenol over CeO_2 and MnO_x

The adsorption of phenol over the single oxide, CeO_2 and MnO_x showed bands due to phenolate species from the dissociative adsorption of phenol. Over CeO_2, upon exposure to the 0.1 M phenol/O_2 water solution, bands were observed to form initially at 1586, 1481 and 1262 cm^{-1} with an additional band at 1234 cm^{-1} forming as the exposure time of the catalyst to the oxygen saturated phenol solution increased (**Fig. S10**). Previous studies reported the ν(CO) vibration of Ce-phenolates at 1273 cm^{-1} for Pd/CeO_2 [7] and for CeO_2 at 1266 cm^{-1} and 1242 cm^{-1} which were assigned to monodentate and bidentate phenolates respectively [8]. Herein, over CeO_2, monodentate phenolate species were observed to form initially (ν(CO) at 1262 cm^{-1}) before bidentate phenolate species (ν(CO) at 1234 cm^{-1}) and with further exposure time, bands were also observed to form in the 1600–1700 cm^{-1} region. Bands in the 1600–1700 cm^{-1} region have also been observed on CeO_2 following low temperature ozonolysis of phenol [8] and also in spectra over vanadia-titania catalysts during the oxidation of benzene wherein these bands were assigned to partial oxidation intermediates and adsorbed quinones (~1680 cm^{-1} to *o*-quinone and ~1660 cm^{-1} to *p*-quinone), respectively [9]. In addition, bands in the 1700 cm^{-1} region have been reported in the photodecomposition of phenol over TiO_2 and assigned to the oxidation of phenol to carboxylic acids such as oxalic acid (bands at the 1715 and 1690 cm^{-1} region due to oxalate species chemisorbed on TiO_2) [10]. The formation of the bands at 1600–1700 cm^{-1}, due to species adsorbed on the surface of the catalyst, indicate oxidation of (bidentate) phenolates to quinones/carboxylic acids (ring opened) over CeO_2 in the conditions used in this ATR study.

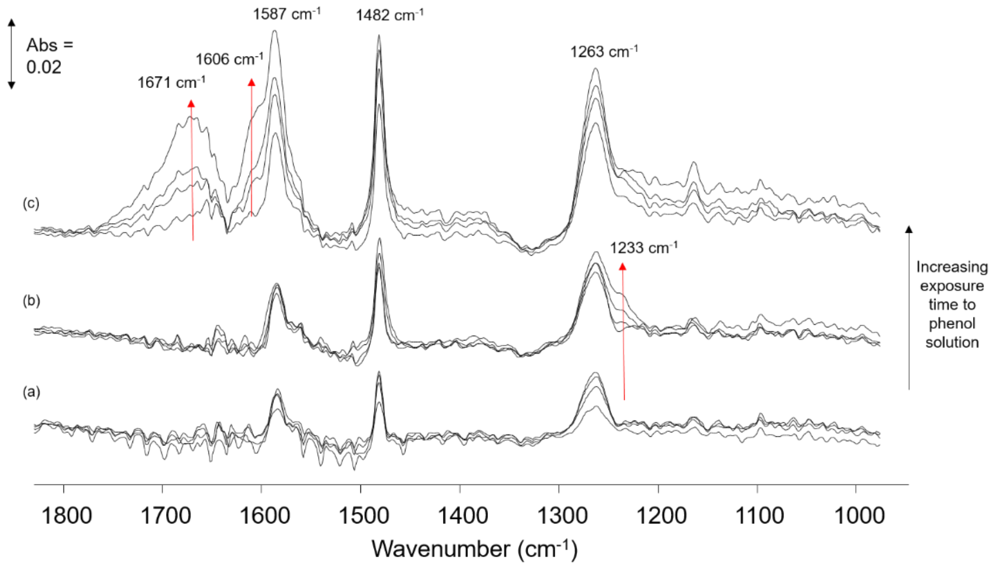

Fig. S10. ATR-IR spectra of 0.1 M phenol/H_2O/O_2 over CeO_2 at 95 °C (a) initial interaction showing formation of monodentate phenolates, (b) formation of bidentate phenolates and (c) partially oxidised products (quinones and acids) under increasing exposure time to the phenol solution. Bands due to water and liquid phase phenol have been subtracted.

Contrastingly, following exposure of MnO_x to the phenol/water/O_2 solution at 95 °C, weak bands due to adsorbed partially oxidised products were observed to form (1672, 1435 and 1368 cm^{-1}) with additional bands in the spectra found at 1592, 1483 and 1232/1205 cm^{-1} assigned to phenolate species adsorbed on the Mn sites (**Fig. S11**) [11]. While the bands at ~1232/1205 cm^{-1} are red-shifted from the *v*(CO) band of Ce-phenolates, bands at comparable positions have been reported for the phenolates formed from oxidation of benzene on manganese oxide catalysts (1236 cm^{-1}) [11] as well on the molybdenum oxide phase in MoO_x/Al_2O_3 (1217 cm^{-1}) with phenolates adsorbed on Al_2O_3 sites in MoO_x/Al_2O_3 giving rise to multicomponent bands at 1295/1271 cm^{-1} [12]. The adsorption of phenol on the vanadium oxide phase in V_2O_5/TiO_2 and V_2O_5/ZrO_2 catalysts also exhibited a phenolate *v*(CO) band at 1210 cm^{-1} with the phenolates on TiO_2 or ZrO_2 sites again occurring at higher wavenumbers (1280 cm^{-1}) [9] while for V_2O_5/Al_2O_3, bands due to surface phenolates were formed at 1234/1215 cm^{-1} in the oxidation of 1,2-dichlorobenzene [13]. Bands in this region have also been observed for the adsorption of hydroquinone on TiO_2 formed from the photooxidation of 4-chlorophenol [14] and therefore could be due to formation of dihydroxy phenols on MnO_x, which have been proposed as primary intermediates in the CWO of phenol [15].

For both CeO_2 and MnO_x, the strength of adsorption of the oxidised products/phenolate species was investigated by switching from the 0.1 M phenol/O_2/water solution to a water/O_2 solution while at 95 °C. For CeO_2, bands due to the phenolate species decreased in intensity under the water/O_2 flow with no change in intensity of the bands due to the partial oxidation products indicating that the partially

oxidised products were more strongly adsorbed on CeO_2 than the phenolate species (**Fig. S12**). Over MnO_x, there was no loss of intensity of the phenolate bands under the water/O_2 flow at 95 °C whilst the bands due to partially oxidised products decreased in intensity showing phenolates to be more strongly adsorbed than the partially oxidised products on MnO_x while on CeO_2 partially oxidised products were more strongly adsorbed than phenolates (**Fig. S13**).

In salt water, for CeO_2, bands due to monodentate and bidentate phenolates were observed. These bands were of comparable intensity in salt water, while in water, the monodentate phenolates were significantly more intense (**Fig. S12**). In addition, partial oxidation products were observed to form on the catalyst in the ATR spectra of phenol in salt water over CeO_2, however, they were of lower intensity than in water. No homogeneous reaction occurred in water (phenol removal was ~2%) and the bands due to partial oxidation products were strongly bound to $MnCeO_x$ and CeO_2 catalysts being retained after a water or salt water wash and so if an increased concentration of partial oxidation products were formed from the homogenous conversion of phenol to intermediates, increased adsorption of the intermediates along with reduced phenolates would be expected in the salt water spectra which was not observed.

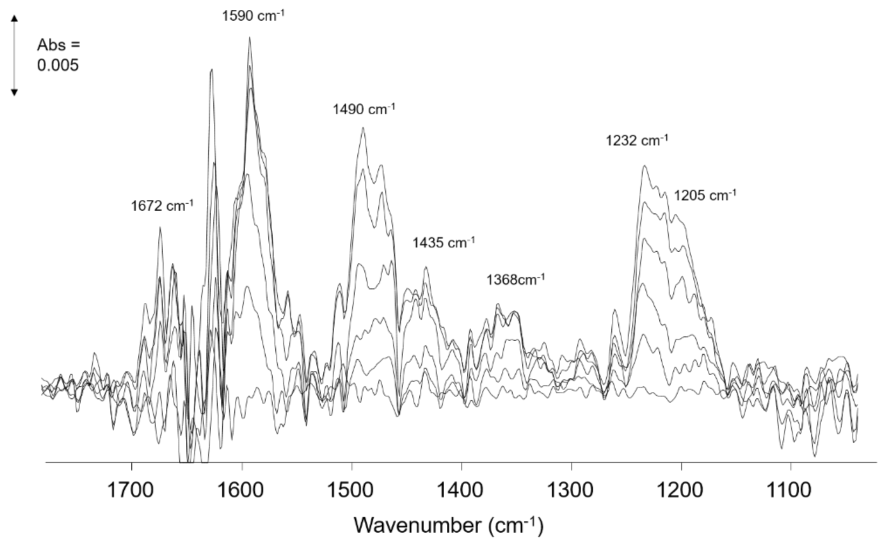

Fig. S11. ATR-IR spectra of 0.1 M phenol/H_2O/O_2 over MnO_x at 95 °C under increasing exposure time to the phenol solution. Bands due to water and liquid phase phenol have been subtracted.

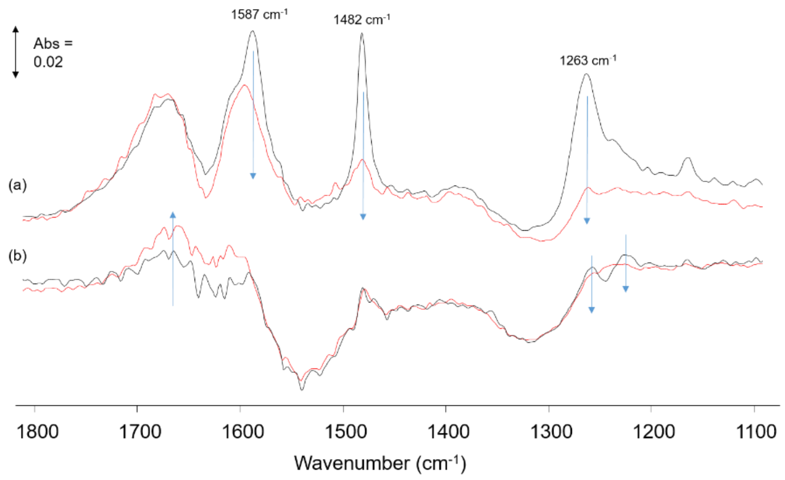

Fig. S12. ATR-IR spectra of CeO_2 (a) black spectrum = 0.1 M phenol in water/O_2 at 95 °C and red spectrum = after water/O_2 flow at 95 °C and (b) black spectrum = 0.1 M phenol in salt water/O_2 at 95 °C and red spectrum = after salt water/O_2 flow at 95 °C. Bands due to water and liquid phase phenol have been subtracted.

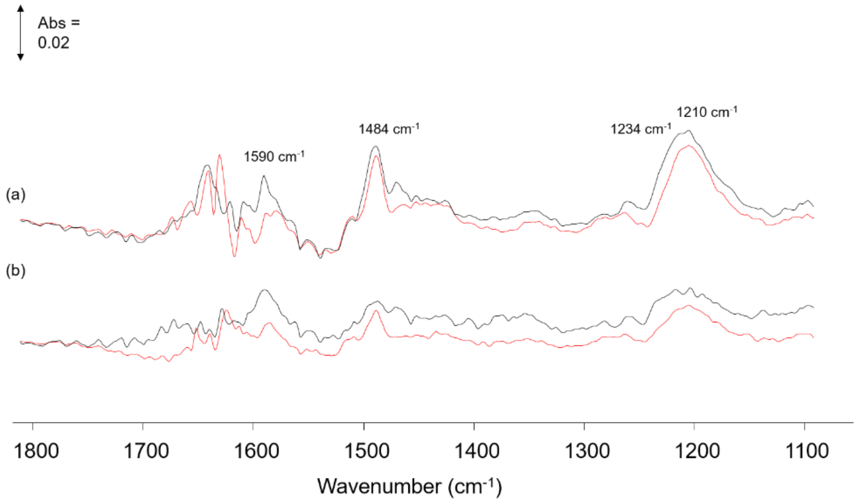

Fig S13. ATR-IR spectra of MnO_x (a) black spectrum = 0.1 M phenol in salt water/O_2 at 95 °C and red spectrum = after salt water/O_2 flow at 95 °C and (b) black spectrum = 0.1 M phenol in water/O_2 at 95 °C and red spectrum = after water/O_2 flow at 95 °C. Bands due to water and liquid phase phenol have been subtracted.

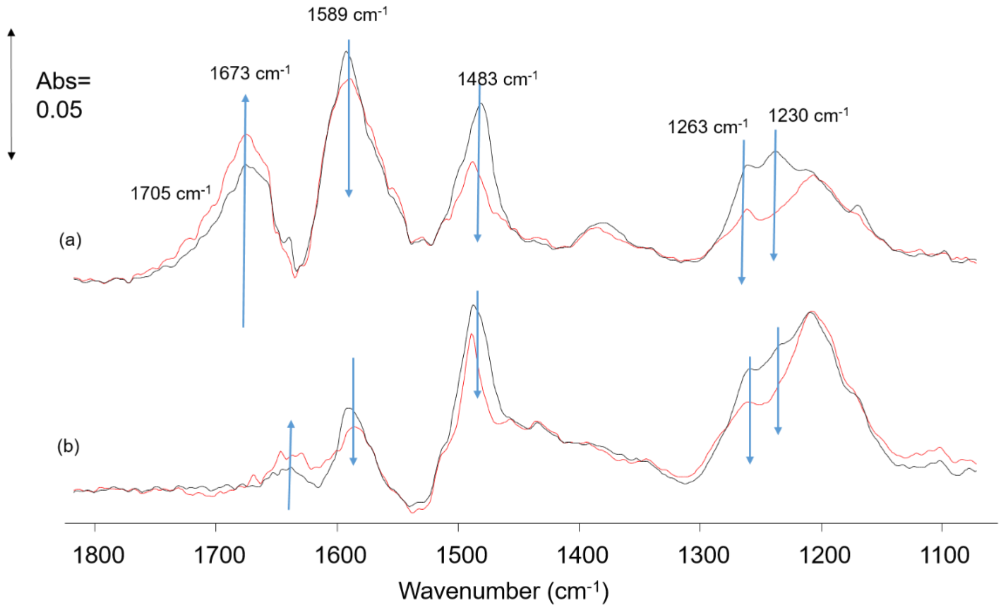

Fig. S14. ATR-IR spectra of $MnCeO_x$ (a) black spectrum = 0.1 M phenol in water/O_2 at 95 °C and red spectrum = after water/O_2 flow at 95 °C and (b) black spectrum = 0.1 M phenol in salt water/O_2 at 95 °C and red spectrum = after salt water/O_2 flow at 95 °C. Bands due to water and liquid phase phenol have been subtracted.

8. Recycle reactions and regeneration of used $MnCeO_x$

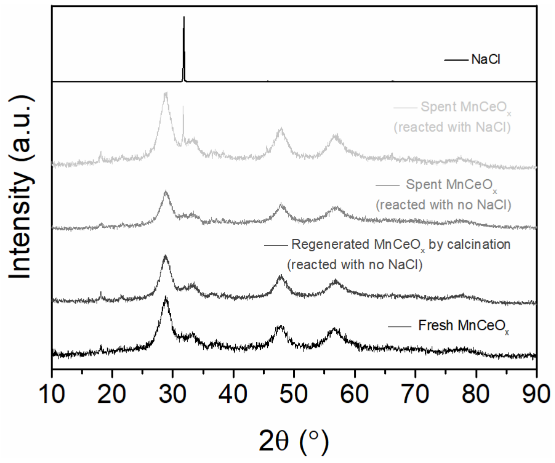

Fig.S15. XRD patterns of fresh and spent $MnCeO_x$ catalysts.

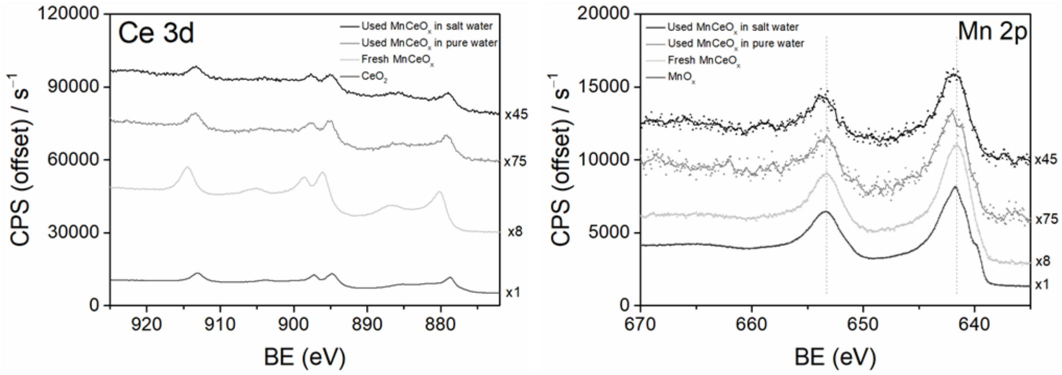

Fig. S16. XPS spectra of Ce 3d and Mn 2p of spent $MnCeO_x$ recovered from a reaction at 110 °C and 0.5 MPa O_2

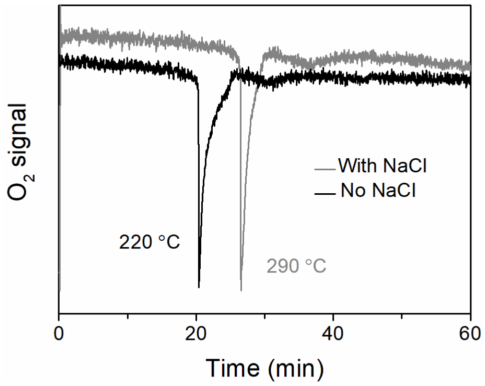

Fig. S17. TPO MS profiles of spent $MnCeO_x$ catalysts (conditions: C_{phenol} = 1.0 g L^{-1}, P_{O2} = 0.5 MPa, 2.0 g L^{-1} $MnCeO_x$, *t* = 2 h).

Table S2

Desorption properties of carbonaceous deposits in spent $MnCeO_x$ catalysts analysed by TPO-MS (conditions: C_{phenol} = 1.0 g L^{-1}, P_{O2} = 0.5 MPa, 2.0 g L^{-1} $MnCeO_x$, *t* = 2 h).

	CO_2 signal		H_2O signal	
	NaCl: 0 g L^{-1}	NaCl: 200 g L^{-1}	NaCl: 0 g L^{-1}	NaCl: 200 g L^{-1}
Peak area*	98	90	14	13

*Peak areas were integrated by Origin. It should be noted that TPO measurements in the same apparatus are known to produce sharp, single peaks, so the high temperature shoulder is not an artefact and is included in the integrated area. Not including it would only increase the difference in CO_2 areas and so still supporting the observations made.

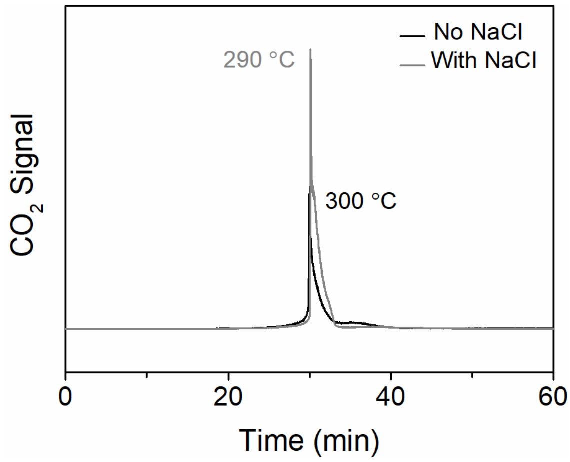

Fig. S18. TPO-MS profiles of used MnCeOx catalysts (conditions: C_{phenol} = 1.0 g L^{-1}, P_{O2} = 0.5 MPa, $C_{catalyst}$ = 1.0 g L^{-1}, t = 24 h).

$$Catalytic\ activity\ recovery = \frac{\chi_{regenerated}}{\chi_{fresh}} \times 100\% \qquad \text{(S1)}$$

where $\chi_{regenerated}$ and χ_{fresh} refer to the phenol removal by the fresh catalyst and the phenol removal by the regenerated catalyst.

Table S3

pH values and metal leaching in the treated water samples (conditions: C_{phenol} = 1.0 g L^{-1}, P_{O2} = 0.5 MPa, 1.0 g L^{-1} $MnCeO_x$, t = 2 h).

	Treated water samples	
	NaCl: 0 g L^{-1}	NaCl: 200 g L^{-1}
pH	5.3	5.7
Mn leaching	0.6 *wt*%	0.5 *wt*%
Ce leaching	0	0

References

(1) Mishra, V. S.; Mahajani, V. V.; Joshi, J. B. Wet Air Oxidation. *Ind. Eng. Chem. Res.* **1995**, *34* (1), 2-48.
(2) Appiani, E.; Ossola, R.; Latch, D. E.; Erickson, P. R.; McNeill, K. Aqueous singlet oxygen reaction kinetics of furfuryl alcohol: effect of temperature, pH, and salt content. *Environ Sci Process Impacts* **2017**, *19* (4), 507-516.
(3) Kumar, A. Salt effects on Diels-Alder reaction kinetics. *Chem Rev* **2001**, *101* (1), 1-19.
(4) Breslow, R. Hydrophobic effects on simple organic reactions in water. *Accounts of Chemical Research* **2002**, *24* (6), 159-164. Grebel, J. E.; Pignatello, J. J.; Mitch, W. A. Effect of halide ions and carbonates on organic contaminant degradation by hydroxyl radical-based advanced oxidation processes in saline waters. *Environ Sci Technol* **2010**, *44* (17), 6822-6828.
(5) Hu, B.; Chen, C.-h.; Frueh, S. J.; Jin, L.; Joesten, R.; Suib, S. L. Removal of Aqueous Phenol by Adsorption and Oxidation with Doped Hydrophobic Cryptomelane-Type Manganese Oxide

(K−OMS-2) Nanofibers. *J. Phys. Chem. C* **2010**, *114* (21), 9835-9844. Arena, F.; Negro, J.; Parmaliana, A.; Spadaro, L.; Trunfio, G. Improved MnCeOxSystems for the Catalytic Wet Oxidation (CWO) of Phenol in Wastewater Streams. *Ind. Eng. Chem. Res.* **2007**, *46* (21), 6724-6731. Arena, F. Multipurpose composite MnCeO x catalysts for environmental applications. *Catal. Sci. Technol.* **2014**, *4* (7), 1890-1898.
(6) Lair, A.; Ferronato, C.; Chovelon, J.-M.; Herrmann, J.-M. Naphthalene degradation in water by heterogeneous photocatalysis: an investigation of the influence of inorganic anions. *J. Photochem. Photobiol. A* **2008**, *193* (2-3), 193-203. Arafat, H. A.; Franz, M.; Pinto, N. G. Effect of salt on the mechanism of adsorption of aromatics on activated carbon. *Langmuir* **1999**, *15* (18), 5997-6003.
(7) Nelson, N. C.; Manzano, J. S.; Sadow, A. D.; Overbury, S. H.; Slowing, I. I. Selective hydrogenation of phenol catalyzed by palladium on high-surface-area ceria at room temperature and ambient pressure. *ACS Catalysis* **2015**, *5* (4), 2051-2061.
(8) Mariey, L.; Lamotte, J.; Lavalley, J.; Tsyganenko, N.; Tsyganenko, A. Low temperature FTIR spectroscopic study of ozone interaction with phenol adsorbed on silica and ceria. *Catalysis letters* **1996**, *41* (3-4), 209-211.
(9) Miyata, H.; Ohno, T.; Hatayama, F. FTIR studies of the interaction of aromatic hydrocarbons with vanadium oxide layered on ZrO2 and TiO2. *Journal of the Chemical Society, Faraday Transactions* **1995**, *91* (19), 3505-3510. Centi, G.; Trifiró, F. *New Developments in Selective Oxidation*; Elsevier, 1990.
(10) Mino, L.; Zecchina, A.; Martra, G.; Rossi, A. M.; Spoto, G. A surface science approach to TiO2 P25 photocatalysis: An in situ FTIR study of phenol photodegradation at controlled water coverages from sub-monolayer to multilayer. *Appl. Catal. B-Environ.* **2016**, *196*, 135-141.
(11) Wang, J.; Zhao, H.; Song, J.; Zhu, T.; Xu, W. Structure-Activity Relationship of Manganese Oxide Catalysts for the Catalytic Oxidation of (chloro)-VOCs. *Catalysts* **2019**, *9* (9), 726.
(12) Popov, A.; Kondratieva, E.; Mariey, L.; Goupil, J. M.; El Fallah, J.; Gilson, J.-P.; Travert, A.; Maugé, F. Bio-oil hydrodeoxygenation: Adsorption of phenolic compounds on sulfided (Co) Mo catalysts. *J. Catal* **2013**, *297*, 176-186.
(13) Krishnamoorthy, S.; Amiridis, M. D. Kinetic and in situ FTIR studies of the catalytic oxidation of 1, 2-dichlorobenzene over V2O5/Al2O3 catalysts. *Catal. Today* **1999**, *51* (2), 203-214.
(14) Stafford, U.; Gray, K. A.; Kamat, P. V.; Varma, A. An in situ diffuse reflectance FTIR investigation of photocatalytic degradation of 4-chlorophenol on a TiO2 powder surface. *Chemical physics letters* **1993**, *205* (1), 55-61.
(15) Santos, A.; Yustos, P.; Cordero, T.; Gomis, S.; Rodriguez, S.; Garcia-Ochoa, F. Catalytic wet oxidation of phenol on active carbon: stability, phenol conversion and mineralization. *Catal. Today* **2005**, *102*, 213-218.
